# The First Endogenous Herpesvirus, Identified in the Tarsier Genome, and Novel Sequences from Primate Rhadinoviruses and Lymphocryptoviruses

**DOI:** 10.1371/journal.pgen.1004332

**Published:** 2014-06-19

**Authors:** Amr Aswad, Aris Katzourakis

**Affiliations:** Department of Zoology, University of Oxford, Oxford, Oxfordshire, United Kingdom; University of Utah School of Medicine, United States of America

## Abstract

*Herpesviridae* is a diverse family of large and complex pathogens whose genomes are extremely difficult to sequence. This is particularly true for clinical samples, and if the virus, host, or both genomes are being sequenced for the first time. Although herpesviruses are known to occasionally integrate in host genomes, and can also be inherited in a Mendelian fashion, they are notably absent from the genomic fossil record comprised of endogenous viral elements (EVEs). Here, we combine paleovirological and metagenomic approaches to both explore the constituent viral diversity of mammalian genomes and search for endogenous herpesviruses. We describe the first endogenous herpesvirus from the genome of the Philippine tarsier, belonging to the *Roseolovirus* genus, and characterize its highly defective genome that is integrated and flanked by unambiguous host DNA. From a draft assembly of the aye-aye genome, we use bioinformatic tools to reveal over 100,000 bp of a novel rhadinovirus that is the first lemur gammaherpesvirus, closely related to Kaposi's sarcoma-associated virus. We also identify 58 genes of *Pan paniscus lymphocryptovirus 1*, the bonobo equivalent of human Epstein-Barr virus. For each of the viruses, we postulate gene function via comparative analysis to known viral relatives. Most notably, the evidence from gene content and phylogenetics suggests that the aye-aye sequences represent the most basal known rhadinovirus, and indicates that tumorigenic herpesviruses have been infecting primates since their emergence in the late Cretaceous. Overall, these data show that a genomic fossil record of herpesviruses exists despite their extremely large genomes, and expands the known diversity of *Herpesviridae*, which will aid the characterization of pathogenesis. Our analytical approach illustrates the benefit of intersecting evolutionary approaches with metagenomics, genetics and paleovirology.

## Author Summary

*Herpesviridae* is a family of DNA viruses that have characteristically large and complex genomes. This defining feature is also responsible for bioinformatic challenges that complicate herpesvirus genomics, and why an endogenous herpesvirus remains elusive. Given that several species of herpesvirus are clinically relevant to humans, there is a pressing demand for techniques capable of generating and managing large quantities of herpesvirus genome data. This is coupled with a need to explore herpesvirus diversity in order to understand pathogenesis within an evolutionary context. Lessons from the study of ancient viral integrations have also highlighted the need to include information offered by paleoviruses. Using perspectives from paleovirology and metagenomics, we identify three herpesviruses within the genome data of their primate hosts, including the first endogenous herpesvirus. All three viruses are closely related to important human pathogens and two of them are entirely new species. Both comparative molecular biology and evolutionary analysis were applied to examine our results for their clinical relevance. Furthermore, we demonstrate how this analytical approach was also used for the data collection itself, by treating nucleotide databases in their entirety as a single metagenomic resource.

## Introduction

Herpesviruses are double-stranded DNA viruses that maintain pervasive life-long infections that are intimately linked to their hosts' biology. They exhibit a long history of host co-evolution [1]–[3], resulting in viruses that are highly adapted to their natural host. This specialization is responsible for an intricately balanced host/virus relationship, resulting in characteristically asymptomatic infections [4]. Disruption to this balance can occur during periods of immunosuppression, resulting in pathogenesis ranging in severity from mild blisters to cancer [4], [5]. Imbalance can also result in disease if a cross-species transmission introduces the virus to a new host [6]. This contrast between a benign state and potentially fatal pathogenesis means that herpesviruses represent a unique system to study the mechanisms of disease development, as well as the influences of co-evolution on viral emergence. A thorough appreciation of herpesvirus biology and evolutionary dynamics, however, requires access to whole genomic data, as well as strategies for novel herpesvirus discovery. The complexity that makes herpesviruses an ideal study group is also responsible for exacerbating the challenge of sequencing them. Although hundreds of species have been recorded, their genome size and complexity (often including host derived sequences), ensures that only a fraction of these have been sequenced, which limits their analytical potential.

In recent years, more detailed macroevolutionary studies of viruses have become possible via endogenous viral elements (EVEs) that are ancient in origin [7]. These genomic relics are the result of accidental host germline integration events by viruses that eventually reach population fixation. In essence these EVEs ‘fossilize’ in the species' genome and are thereafter transmitted vertically as part of the host gene repertoire. By virtue of their replication mechanism, retroviruses represent the vast majority of this viral fossil record, as they necessarily integrate into the host genome [8]. This relative abundance is due in part to the difficulty in searching for EVEs from other groups, because identifying fossils of larger viral genomes with complicated natural histories is far more bioinformatically challenging. Herpesviruses are emblematic of these difficulties due to their low sequence conservation, lack of consistent gene order and evidence of host-gene recombination [3]. Indeed, in the most comprehensive EVE survey conducted to date, herpesviruses were excluded from the search [7].

Despite remaining elusive thus far, there is nonetheless reason to suspect the existence of a herpesvirus EVE (HEVE), as several herpesviruses are known to undergo strategic somatic genome integration to establish their latent state. Known examples include Epstein-Barr virus (EBV) [9], [10], Marek's disease virus (MDV) [11]–[13] and human herpesvirus 6 (HHV6) [14], [15], although integrated EBV viruses represent a small fraction of infected cells. HHV6 is particularly noteworthy as it has been shown that the virus is capable of germline integration that has led to speculation that HHV6 is an EVE at an early stage of the fossilization process, which has yet to reach an appreciable frequency in the population [7]. Less than 1% of humans (UK and US data) carry a heritable HHV6 (ciHHV6) integrated into telomeric regions of various chromosomes [15], [16]. Both HHV6 and MDV genomes possess host satellite telomeric repeats (TMRs) within the repeat regions found at either end of their genomes. Despite being controversial for many years, a large body of work has established the telomere-specific integration of both HHV6 and MDV that occurs via a poorly characterized process of homologous recombination between viral and host TMRs [10], [11], [15].

Paleovirology as a discipline has only become possible due to innovations in sequencing technology that allow the rapid sequencing of whole genomes. The same advances have also unlocked avenues in metagenomics, which are especially valuable in clinical settings such as mapping the human virome [17]. Paleovirology can be seen as contributing to this effort, if we consider the virome as not only the viruses that are either in or on species, but also the collection of viruses embedded in their genome. Indeed the technical challenges are not dissimilar, as in both cases the objective is to identify and characterise minute proportions of viral nucleic acid from data that is overwhelmed with host sequences. The only difference is that unlike host genome sequencing efforts, metagenomics applied to the human virome actively welcomes ‘contaminants’, as they are the subjects of study. Similar to metagenomic techniques, though admittedly with a narrower scope, viral identification has been achieved by deep sequencing of clinical samples that subsequently filter out host sequences using a reference [18]. This approach is limited, however, to situations where reference sequences exist for the host genome and often also that of the virus. Although this issue is also a limitation in paleovirological surveys, since viral references are used to probe genome records, the sequences need only be similar enough for initial screening. Furthermore, *a priori* knowledge of the host genome is not necessary for EVE identification, because paleovirology employs evolutionary techniques as part of the process of characterising sequences. As a consequence of their rarity, new EVEs are sought by querying entire databases, rather than individual genomes. This maximises the chance of EVE detection, treating the nucleotide content of databases as though they were a single resource, or metagenome.

Recognising that paleovirology and viral metagenomics share many of the same goals and techniques, we set out to establish the benefits of approaching viromics with this interdisciplinary perspective in mind. This means that the ultimate objective was to explore viral diversity through bioinformatic means alone, as a proof-of-principle that demonstrates the hidden value of online genomic databases. Because HHV6 is the only known herpesvirus capable of germline chromosomal integration, we hypothesized that a closely related HEVE should be detectable using HHV6 as an initial probe. As a result, we describe novel *Herpesviridae*-like (HVL) sequences from the genome of the aye-aye (*Daubentonia madagascariensis)*, bonobo (*Pan paniscus*) and Philippine tarsier (*Tarsius syrichta*). We recognise the viral sequences of *P. paniscus* as the previously described *Pan paniscus lymphocryptovirus 1* (PpanLCV1), for which only 3,190 bp had been identified [19]. Our analyses demonstrate that two of these (*T. syrichta* and *D. madagascariensis*) sequence sets represent partial genomes of entirely new virus species, in mammals that we did not know to be herpesvirus hosts. Most notably our analyses reveal that the *T. syrichta* sequences represent the first endogenous herpesvirus whose ancestral exogenous relative closely resembled HHV6. Each set of sequences we identified is referred to throughout the text as PpanHVL (*Pan paniscus Herpesviridae*-like), DmadHVL (*Daubentonia madagascariensis Herpesviridae*-like) and TsyrHVL (*Tarsius syrichta Herpesviridae*-like). The TsyrHVLs are additionally referred to as the tarsier herpesvirus endogenous viral element (THEVE), when discussing the endogenous virus.

## Results

We used consensus sequences of 33 NCBI viral protein clusters (table S1) to initially screen the mammalian genomes in the WGS database, which includes 14 primate genomes from 9 species. Of the 114 hits, we shortlisted those of bonobo, aye-aye and Philippine tarsier for further investigation, with 76 viral hits reported on 22 contigs (results summary in table S2). We identified viral hits to EBV in human, which is a well-characterised and fully sequenced herpesvirus, and we therefore did not investigate them further. Each of the shortlisted genomes (table 1) were then searched in more detail using a larger query set of all proteins from fully sequence viral genomes of genera that showed similarity to the primates in the first BLAST round. From the resulting hit list, we performed a reciprocal BLASTx search of 165 contigs against viral proteins in the NCBI nr database, thereby eliminating 54 false positives (table S3). Contigs were visualised by creating conceptual schematics of each sequence set by mapping the homologous regions for each sequence against an appropriate reference genome. Herpesvirus genomes fall into 6 general layouts with respect to their unique coding regions in addition to the number and orientation of repetitive blocks [4]. Although it is not a rule, herpesviruses of the same lineage tend to exhibit the same overall genome architecture and closely related herpesviruses (e.g., belonging to the same genus) tend to maintain a similar gene set and organisation (figure S1) [4]. We therefore used viruses closely related to each HVL sequence set as a guide to mapping and visualising the contigs.

**Table 1:** Genomes containing Herpesvirus-like sequences.

		*Pan paniscus*	*Daubentonia madagascariensis*	*Tarsius syrichta*
WGS Genome records	Sex/animal name	Female ‘Ulindi’	Male ‘Goblin’	Female (name unkown)
	Institute	Leipzig Zoo	Duke Lemur Centre	Duke Lemur Centre
	NCBI BioSample ID	SAMEA1029457/SAMEA1029458	SAMN00690380	SAMN02445010
	Tissue	Blood	Liver (necropsy)	N/A
	Coverage	26×	38×	48×
	WGS project ID	AJFE0	AGTM0	ABRT0
	Sequencing platform	GS FLX (454)	Illumina GA II	Sanger/Illumina mixed
	Total sequence length	2,869,206,676	2,855,365,987	3,453,847,770
	Total assembly gap length	143,237,068	0	48,109,210
	Gaps between scaffolds	0	N/A	0
	Number of scaffolds	10,868	N/A	337,188
	Scaffold N50	10,124,892	N/A	401,181
	Number of contigs	121,235	3,231,305	492,902
	Contig N50	66,775	3,653	38,165
	Total number of chromosomes and plasmids	1	0	0
	Reference/credits (According to WGS record)	[76]/Kay Prüfer *et. al.*	[77]/George H. Perry *et. al.*	Wesley Warren, Richard K. Wilson & the Genome Institute, Washington Univ. School of Medicine

Details of the genome records that were investigated for viral sequences. Information was obtained from the NCBI assembly website: http://www.ncbi.nlm.nih.gov/assembly/. N50 is the contig length at which all contigs of that length or longer represent 50% of the total of the lengths of all contigs.

To determine the taxonomy and evolutionary relationships of these HVLs to *Herpesviridae*, a series of phylogenetic trees were reconstructed. Previous phylogenetic studies have reproducibly established the common evolutionary origin for each of the subfamilies in *Herpesviridae* [2], [4]. Because of the complicated natural history of herpesvirus genomes, single-gene trees do not necessarily reflect the true evolutionary relationships. The dependence of phylogenetic accuracy on gene availability poses a challenge for novel virus characterization, because new sequences need to be compared against the greatest range of available diversity possible. Doing this, however, necessitates the inclusion of partially sequenced herpesviruses, many of which are identified by only a single gene, or less. This trade off between phylogenetic accuracy and detail was taken into consideration when investigating the HVLs, and we employed multiple phylogenetic strategies, rather than depending on a single tree, to arrive at the best possible characterisation.

### A herpesvirus endogenous viral element in *Tarsius syrichta*

**Figure 1:**
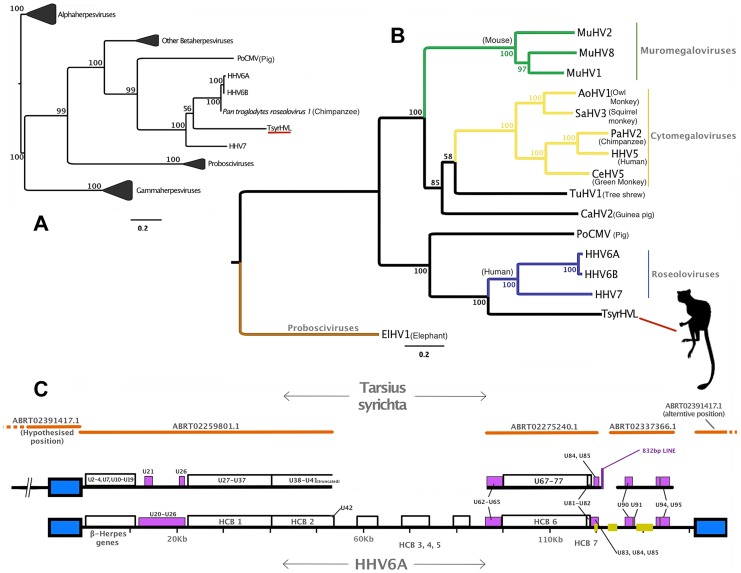
Phylogenetic and genomic analysis of the tarsier endogenous herpesvirus. Panel A: DNA polymerase tree showing the placement of the TsyrHVL sequence within the *Betaherpesvirinae*. Only the lineage leading to the node including TsyrHVL is shown and the rest are collapsed for clarity, and the size of the collapsed clade is arbitrary. Panel B represents the phylogeny reconstructed from a concatenated amino acid alignment of 6 core genes (terminase, large tegument, uracil-DNA glycosylase, kinase, capsid protein and helicase). Unclassified betaherpesviruses are shown as black branches, whereas those belonging to defined genera are indicated in colour. The rooting at *Proboscivirus* was determined according to the phylogeny in panel A. Numbers at each node in both Panel A and B represent bootstrap support. Panel C shows a schematic of the tarsier sequences mapped to HHV6 as a reference. Orange lines indicate wgs contigs obtained from NCBI and GenBank IDs are annotated. Contig ABRT02391417.1 is represented on both sides, since it consists entirely of the DR region, although it aligns with ABRT02259801.1 with only 5 differences, and both placements are plausible. Blue box indicates the virus' terminal direct repeat (DR) regions. Yellow boxes represent the major internal repeat regions. Because the genomes are so large, it is not feasible to represent the complete coding content. Instead major herpesvirus core blocks (HCB) are indicated (as in reference [5]), and genes that are relevant to discussion points in the main text are also annotated. Abbreviations for Panel A and B are THEVE: Tarsier Herpesvirus Endogenous Viral Element, HHV6A/6B/7/5: *human herpesvirus 6A/6B/7/5*, MuHV1/2/8: *Murid herpesvirus 1/2/8*, AoHV1: *Aotine herpesvirus 1*, SaHV3: *Saimiriine herpesvirus 3*, PaHV2: *Panine herpesvirus 2*, CeHV5: *Cercopithecine herpesvirus 5*, TuHV1, PoCMV: *Porcine cytomegalovirus*.

**Figure 2:**
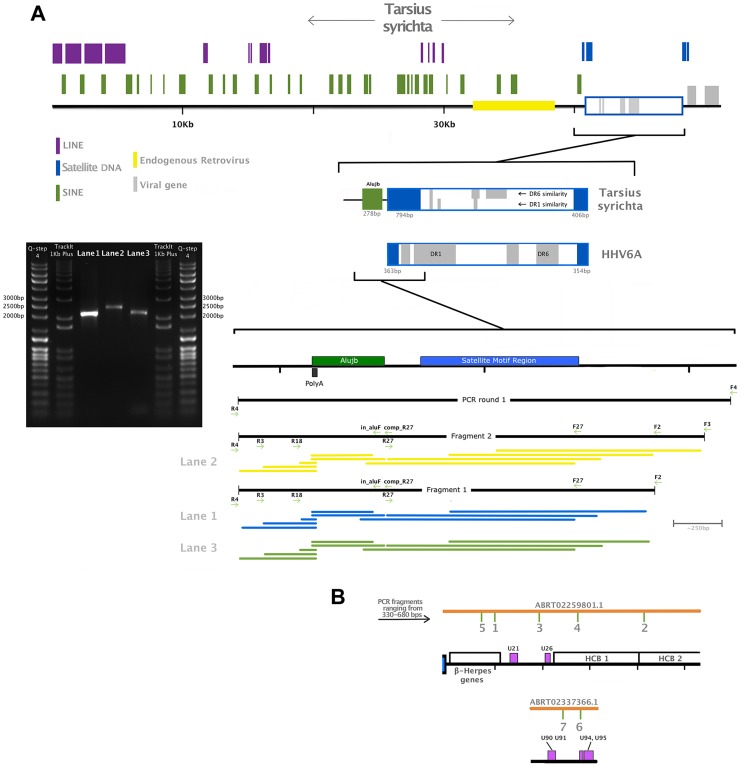
Validation of the endogenous status of TsyrHVLs. Panel A is a close up of contig ABRT02391417.1, showing RepeatMasker-detected repetitive elements, an endogenous retrovirus and the satellite DNA telomeric repeat motif (TTAGGG)n that is characteristic of chromosomally integrated HHV6. A zoomed-in representation of the junction is also depicted, with regions highlighted according to the colour key, as well as a map of primers used for the amplification and location of sequenced fragments. Two independent PCR reactions were run in the first instance using the primer pair F4/R4 and genomic DNA, which was semi-nested in the second round of amplification. A single fragment using primers F2/R4 was obtained from both first-round amplicons, and a larger fragment from primer F3/R4 was possible from one of them. A 1% agarose gel electrophoreses is shown indicating the approximate size of the fragments: F2/R4 amplified a 2,034 bp fragment shown in Lane 1 and 3, while the F3/F4 fragment was 2,277 bp (Lane 2). Each fragment was sequenced using all visible primers (F4 was only possible for the largest amplicon). The coverage map indicates the sequence obtained from each primer, trimmed for quality. The final contig after quality trimming was 2,159 bp, and included 5 nucleotide differences and 2 indels relative to the tarsier genome record. A proportion of these will be true polymorphisms, while others may have resulted from polymerase error during either sequencing or amplification, (in both our fragment and the published sequence). Panel B shows the location of small fragments amplified to confirm the presence of the unique viral region.

Phylogenetic evidence indicated that the *Tarsius syrichta* HVL (TsyrHVL) sequences are most closely related to the roseoloviruses (figure 1A/B), and so they were characterized with the HHV6A genome as a reference (figure 1C). Crucially, the TsyrHVL sequences include the junction between the viral-like region and host chromosome, which was necessary to identify it as a *bona fide* integration. Roughly 40.7 kb upstream of the viral junction in contig ABRT02391417.1 shows high sequence similarity to primate genomes and contains characteristic macroscopic features including LINEs, SINEs and a class 1 endogenous retroviral insertion (Figure 2). The junction itself consist of an array of (ATTGGG) telomeric repeats, as has been shown to be the case for chromosomally integrated HHV6 in the telomere [15]. To ensure that the junction was not the result of erroneous assembly, we independently confirmed the validity of the junction via PCR (figure 2) from a different animal than that of the WGS record. It was important to amplify a single, large fragment that crossed the viral/host junction and also included both the viral and host flanks. Given the length of the product sought, we devised a semi-nested PCR strategy to overcome the fact that the DNA used was of low quality. Primer pairs for a 2,363 bp fragment were initially used on genomic DNA, and although no band was visible by gel electrophoresis, low concentration was detected by spectrophotometry. These PCR products were then amplified using semi-nested primers, and a ‘touchdown’ PCR program, yielding high concentrations of the correct products (*see* Materials and Methods).

Similarly to HHV6, TsyrHVL includes a direct repeat (DR) region that contains two TMR motif regions. The TMR at the TsyrHVL/host junction is almost twice as large as its counterpart, which is a more comparable length to those in HHV6 (∼300 bp). This could have resulted from the mechanism of integration, and scenarios that would create a double-length TMR have been hypothesised for HHV6, albeit not with an identical outcome to the TsyrHVL [10]. Contig ABRT02259801.1 overlaps with ABRT02391417.1 by 337 bp. The overlap region consists of 124 bp from the main DR region, followed by the TMR array that continues past the overlap. There are 4 gaps and 1 mismatch that could be the result of assembly error, which occurs most frequently at contig ends. Alternatively, it is possible that ABRT02391417.1 contains the other DR from the other side of the viral integration, and only aligns to ABRT02259801.1 because the TMRs in both DR regions are identical. Both scenarios are valid in the absence of additional information and represented in figure 1C for clarity. In order to estimate the date of integration, we took advantage of the fact that at the time of integration, the right-most TMR in each DR would have been composed of a series of perfect hexanucleotide repeats. Thus, any differences from this repeat structure would have occurred in TsyrHVL due to neutral evolution after its endogenisation. 294 bp from the viral side TMR was used, beginning and ending with an intact TAACCC motif (the other TMR is far too divergent to have been the original perfect repeat). We inferred the maximum likelihood pairwise divergence of the observed TMR from the hypothetical TMR ancestor using the GTR model of substitution, at 0.165 substitutions/site. As we are measuring the divergence from an ancestral sequence rather than the divergence between contemporary repeats, the distance value was converted into time directly rather than being divided by two first. Using the mammalian neutral rate of evolution of 2.2×10^−9^ [20], the insertion is estimated to be ∼76 million years old. Using the estimate for bushbabies of 3×10^−9^ [21], the date is significantly younger at ∼56my, and maybe more accurate given that they are both relatively small prosimians with similar life histories. Nonetheless, both dates are plausible being after the tarsier divergence of ∼80my [22], consistent with the absence of orthologous insertions (thus far) in other primates. The approximate date, however, should be interpreted with caution, since the repetitive nature of the TMR could mean that the pattern and rate of substitution is significantly non-neutral.

Given that the TMR motif is detectable on the same contig that the left-most viral genes are the DR in our data is likely to be the left DR. Unusually however, the orientation of the ATTGGG motif in the TMRs is the reverse complement to the expected, assuming it should be the same as HHV6. This could either mean that the exogenous viral ancestor to the TsyrHVLs is different to HHV6, or else be the result of the integration mechanism. Because the TsyrHVLs have been in the tarsier genome long enough to accumulate many nonsense mutations, we cannot take advantage of certain genomic features of roseoloviruses that may have informed us on the integration event. This includes the fact that the left and right TMR regions within each DR in exogenous roseoloviruses are not identical, and instead are either perfect or imperfect. In the TsyrHVLs, both are degraded and so cannot be distinguished, and other features such as pac1 and pac2 signaling sites are mutated beyond recognition.

A phylogenetic tree was reconstructed using a short region of the conserved Herpesviral gene DNA polymerase in order to capitalize on partially sequenced herpesviruses for taxonomic breadth (Figure 1A). Although this low-resolution phylogeny is not reliable for detailed evolutionary comparison, it established the sub-family placement of TsyrHVL within *Betaherpesvirinae* with strong bootstrap support. A more robust tree of only betaherpesviruses was obtained using a concatenation of six highly conserved core genes (terminase, large tegument, uracil-DNA glycosylase, kinase, capsid protein and helicase, Figure 1B). The *Roseolovirus* genus currently consists of HHV6, HHV7 and *Pan troglodytes roseolovirus 1* and the tarsier sequences group closest to this lineage. Whereas it resolves basally to the roseoloviruses, the genome organization and gene content is almost identical to HHV6, including Roseolovirus-specific genes (e.g. U21 and U85), thereby supporting their designation as a basal member of the group.

Unlike ciHHV6, nearly all genes in TsyrHVL have lost coding capacity through multiple frame-shifts and stop codons. These cannot be sequencing errors as the tarsier genome was sequenced to ∼48× coverage, and is rather characteristic of fossilized EVEs as their evolutionary longevity depends on either being beneficial or nearly neutral to the host [23], [24]. Consequently, unless the EVE is a currently active captured gene, it will drift at the host neutral rate, accumulating nonsense mutations [7], [8] just as appears to have occurred in the TsyrHVLs.

Contig ABRT02259801.1 begins with the satellite motif followed by a short coding gap and similarity to a conserved block of 13 Betaherpesvirus genes (U2–4, U7, U10–U19) that are collinear with HHV6 (figure 1C). U2–4 are related to the US22 gene family that perform functions in replication and pathogenesis [5]. U11 encodes tegument pp150, the major antigen recognised by HHV6-specific IgG [5]. U12 encodes a G-protein coupled receptor whose function is still unclear, but it is a chemokine receptor that might be involved in promoting cell growth and inhibiting apoptosis, as well as promoting cell migration [25]. Following this block in HHV6, there are 6 genes (U20–26) spanning 6835 bp, but only U21 and U26 were detectable by BLAST, in a region that is approximately half the size of the HHV6 equivalent. After this, the TsyrHVL contains similarity to the herpesviral core block 1 (HCB1), which is composed of 11 conserved genes (U27–37). Of these, all but the small capsid protein and DNA packaging protein UL33 appear to have lost coding capacity in TsyrHVL. ABRT02259801.1 ends with most of HCB2, which in HHV6 is composed of U38–U42. Clear similarity can be detected to all but one of these genes, as it ends within U41, most likely due to the draft state of the assembly. This is also true for the TsyrHVL equivalent of HCB3-5, which were not found among the data.

Contig ABRT02275240.1, is mostly composed of HCB6 (genes U67–77) that is preceded by similarity to non-core HHV6 genes U62–65. All TsyrHVL HCB6 genes have lost coding capacity, except U68 and U72 that encode a tegument protein and envelope glycoprotein M. In HHV6, U62 is situated immediately downstream of the first exon of DNA packaging terminase subunit 1 (U60). Although it is absent in the TsyrHVL, the second exon is situated following three disrupted ORFs similar to U63–U65. HCB6 is succeeded by a 2,590 bp coding gap in HHV6A that is only 1,612 bp in TsyrHVL, followed by U79–82. In HHV6A and HHV6B, the next coding sequence would be U83–U85, but the TsyrHVL is more similar to HHV7, in that the chemokine gene is absent. ABRT02275240.1 ends with similarity to U86, the Immediate-Early IE2 gene that is involved in the virus' latent cycle. In HHV6 IE2 is followed by a coding gap that contains one of three major internal repeat regions, which is the most likely cause of assembly failure. The HHV6A genes 90–100 are the last four genes after U86 and encode IE1, membrane protein UL114, U94/*rep*, U95 and the Roseolovirus-specific glycoprotein Q. ABRT02337366.1 exhibits similarity to all except for membrane protein. Interestingly, U94/*rep* is a captured adeno-associated virus gene [5], [26], which means that the TsyrHVL U94/*rep* represents an EVE within an EVE.

In addition to the host/virus junction, we also sequenced 7 fragments from the unique viral region to confirm the presence of the virus beyond the junction (figure 2B). As well as a nucleotide match to the reference sequence, two of the amplified fragments included the characteristic nonsense mutations we would expect to find in endogenous elements. Fragment 1 is a 686 bp partial gene U10, which encodes protein UL31 and contains the same in frame stop codon as in contig ABRT02259801.1. Fragment 2 is 530 bp spanning the whole length of gene U35 and the first 90 bp of U36. Fragment 3 is a 471 bp region the spans the coding gap between genes U17 and U18, and indeed does not appear similar to any proteins. The 325 bp of Fragment 4 includes part of the gene U28, which encodes subunit 1 of ribonucleotide reductase. The last amplicon obtained from ABRT02259801.1 is fragment 5, which is 456 bp of gene U7. Two fragments were amplified from ABRT02337366.1. Fragment 6 is a partial sequence of the captured parvovirus *rep* gene, and includes the first 114 amino acids, as well as 1 of the 4 frame shifts found in ABRT02337366.1, and one in-frame stop codon. Fragment 7 is 462 bp, spanning part of one of 3 internal repeat regions (IR3).

### Exogenous viral genomes in primate genome data

**Figure 3:**
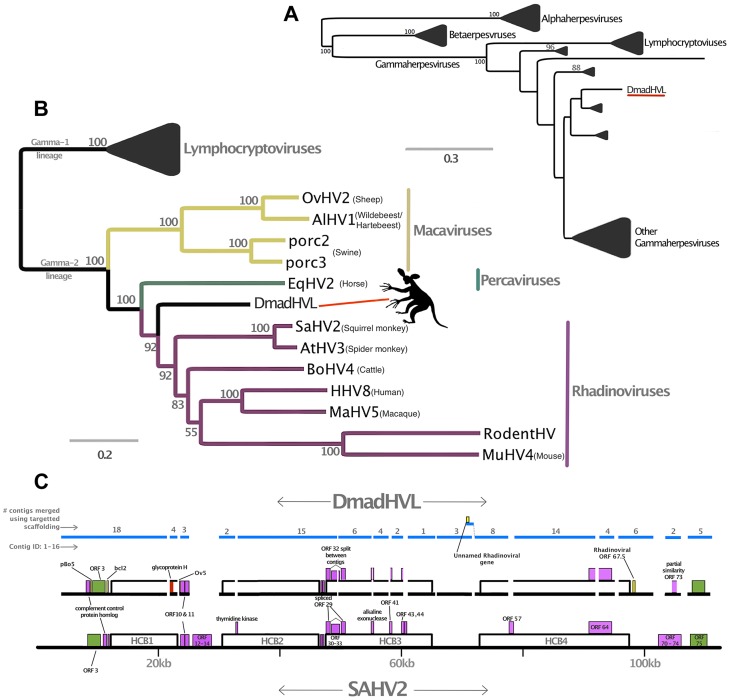
Phylogenetic and genomic analysis of the *Daubentonia madagascariensis* rhadinovirus. Panel A is a maximum likelihood amino acid phylogeny of DNA polymerase, indicating the subfamily placement of the DmadHVL sequence as a gammaherpesvirus. Numbers at each node represent bootstrap support and only those above 50% are shown. Lineages other than those leading to DmadHVL are collapsed for clarity. In Panel B, the tree shown is a maximum likelihood phylogeny estimated using a concatenated alignment of 6 core genes (terminase, large tegument, uracil-DNA glycosylase, kinase, capsid and helicase). Coloured clades represent the different genera within gamma-2 herpesviruses, and bootstrap support is shown for each node. Panel C shows DmadHVL sequences mapped to *Saimiriine herpesvirus 2* (SaHV2) as a guide, and major repeat blocks as well as noteworthy genomic differences and genes discussed in the main text are highlighted in coloured boxes. The green blocks are the FGAM synthase coding sequences, which are found at the termini. The red box annotated as glycoprotein H is presumed to be an assembly error. Pink boxes are discussed genes present in SaHV2, while the yellow ORFs are those found in different viruses. The blue lines indicate the different sequences that are a composite of multiple wgs contigs, the number of which is indicated above each sequence. The composite DmadHVL sequences discussed in the main text are numbered from 1–16 in a left-right direction. The DmadHVL virus genome appears to have a slightly larger region spanning herpes core block (HCB) 3 and HCB4, and so contig 11 is drawn to represent this. The scale of the schematic is approximate. Abbreviations for Panel B are porc2/3: OvHV2: *Ovine herpesvirus 2*, AlHV1: *Alcelaphine herpesvirus 1*, *Porcine lymphotropic herpesvirus 2/3*, EqHV2: *Equine herpesvirus 2*, RodHV: *Rodent herpesvirus peru*, MuHV4: *Murid herpesvirus 4*, AtHV3: *Ateline herpesvirus 3*, SaHV2: *Saimiriine herpesvirus 2*, BoHV4: *Bovine herpesvirus 4*, HHV8: *human herpesvirus 8*, MaHV5: *Macacine herpesvirus 5*.

**Figure 4:**
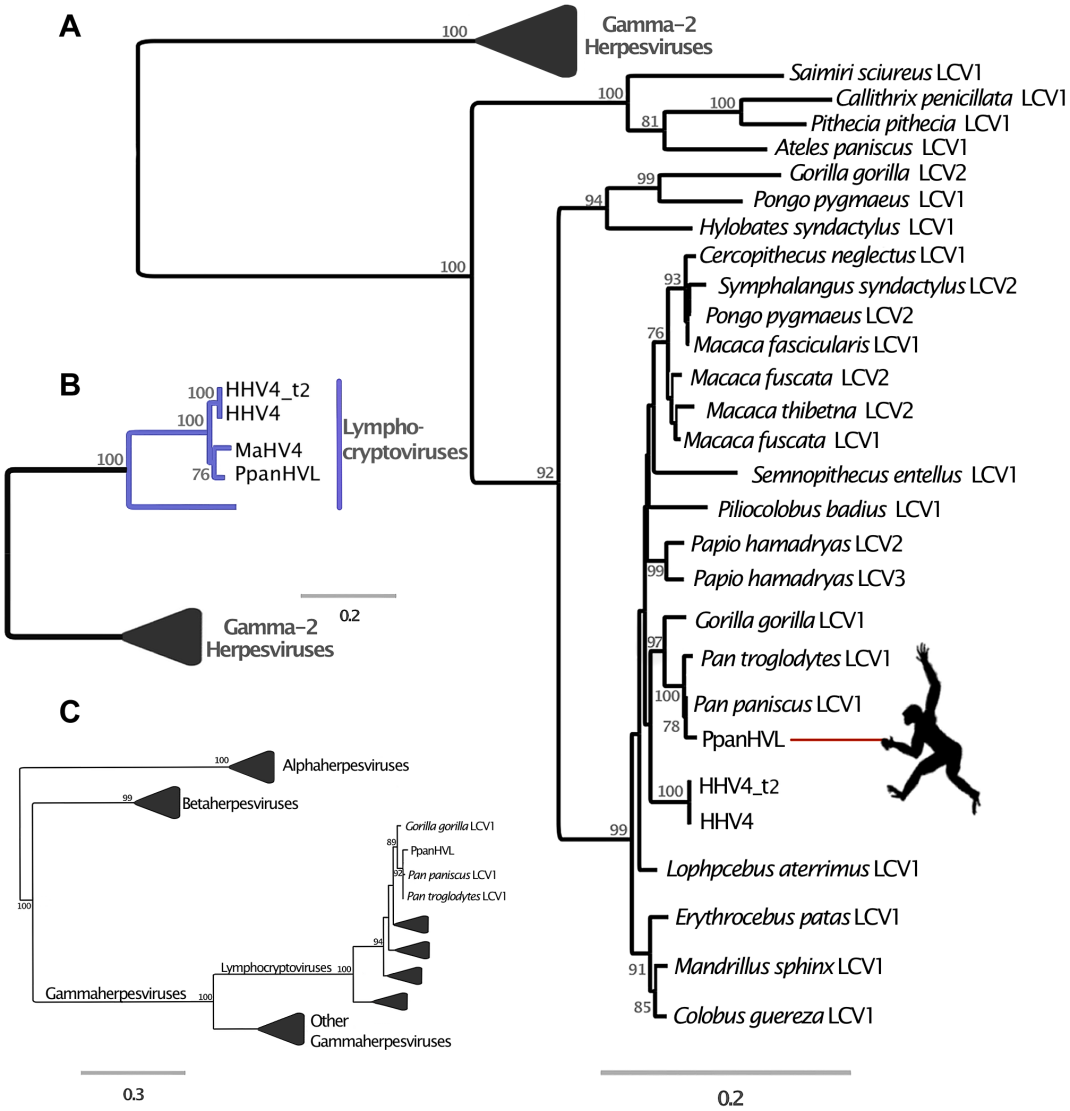
Phylogenetic analyses of *Pan paniscus* lymphocryptovirus. In all panels, the trees depicted represent Maximum likelihood phylogenies reconstructed using different gene sets. Node values represent Bootstrap support but only nodes with over 70% are annotated. The panel A tree was reconstructed using a concatenated amino acid alignment of DNA polymerase and glycoprotein B, for which there are many representative taxa in *Gammaherpesvirinae*. In panel B the tree was reconstructed from a concatenated alignment of 6 core genes (terminase, large tegument, uracil-DNA glycosylase, kinase, capsid and helicase), and in both Panel A and B the gamma-2 lineage is collapsed for clarity. Panel C shows the phylogeny reconstructed using a conserved region of the DNA polymerase gene in order to ascertain the subfamily placement of the PpanHVL sequences. Abbreviations: LCV: *Lymphocryptovirus*, HHV4: *Human herpesvirus 4*, HHV4_t2: *Human herpesvirus 4 type 2*, MaHV4: *Macacine herpesvirus 4*, CaHV3: *Callitrichine herpesvirus 3*.

The evidence from DmadHVL and PpanHVL analyses indicate that they are most likely exogenous viral contaminants, since none of the sequences exhibited non-sense mutations in genes or the acquisition of transposable elements, which are characteristic features of paleoviruses. A DNA polymerase phylogeny established the subfamily placement of both HVLs as gammaherpesviruses (figure 3A and 4C, respectively). There are two main *Gammaherpesvirinae* lineages recognised as γ_1_ and γ_2_ rooted in between [27]. The γ_1_ lineage corresponds to the *Lymphocryptovirus* genus which to date is known to exclusively infect primates and include the PpanHVLs (figure 4A/B). The γ_2_ herpesviruses have a wider host range and the 100% bootstrap support at the base of the lineage place the DmadHVLs within them (figure 3A). A more detailed tree was reconstructed from a concatenated alignment of six core genes (DNA polymerase, glycoprotein B, helicase/primase, ssDNA binding protein, transport protein and major capsid), revealing that the DmadHVLs are neither a member of *Percavirus* nor *Macavirus* (known to infect Perissodactyls and Ruminants respectively), but are rather a sister group to the currently recognised *Rhadinovirus* genus (figure 3B). Despite numerous studies, the topology of the clade has yet to be fully resolved; like the rest of the herpesviruses, it involves broad patterns of co-evolution with their hosts but also frequent deviations that complicate phylogenetic interpretation [2]–[4], [27]–[29]. In *Rhadinovirus*, BoHV4 and MuHV4 are unusual in that their phylogenetic placement is inconsistent with that of their ungulate and rodent clades respectively [2]. Furthermore, there are two lineages of primate rhadinovirus, despite all other primate gammaherpesviruses belonging to the lymphocryptovirus clade. Considering that the topology for the rest of *Gammaherpesvirinae* is congruent with that of the host, the rhadinovirus lineage likely emerged via a cross-species transmission event [2]. According to this hypothesis, the most parsimonious way to explain the topological placement of both BoHV4 and MuHV4 is to invoke a further two species transfer events from primates. Excluding them from the phylogeny leaves a primate rhadinovirus clade with a topology that matches that of their hosts, and the placement of DmadHVLs basal to the group supports this evolutionary scenario. This suggests that the initial transmission would have occurred early in the evolution of primates, prior to the strepsirrhine divergence of ∼87 million years ago [22]. Alternative explanations would require multiple cross species transmissions, potentially once for each of the Rhadinoviral lineages (figure 3B).

A comparison of host and virus branch lengths reveals that old world monkey and ape lymphocryptoviruses are more similar than expected assuming co-evolution and there are no other instances of herpesvirus sequences from different natural hosts being so similar [30]. The topology of the second lineage has been much more difficult to resolve and deviates from the co-speciation expectation, but has nonetheless been closely scrutinised in previous studies and split into multiple genera [31]. The bonobo sequences clearly group within the genus *Lymphocryptovirus* (figure 4B), the type species of which is the human pathogen human herpesvirus 4 commonly known as EBV. The sequence of glycoprotein B and DNA polymerase have already been identified for a bonobo lymphocryptovirus, and the *Pan paniscus* HVLs (PpanHVLs) sequences in this study appear to be the same virus considering the phylogeny in figure 4A and when comparing particular genes. For example, the nucleotide sequence of the glycoprotein B gene is 97 and 98% identical to the corresponding chimpanzee and bonobo sequence, respectively, compared to 89% against EBV. In total, the contigs characterised here extended the genomic sequence for *Pan paniscus lymphocryptovirus 1* (panLCV1) by over 78 kb. Although nearly 95% of the adult human population is infected with EBV, we can be confident that this is not a human contaminant because of the comparatively higher sequence similarity of the PpanHVLs to panLCV1 than to EBV. This is further supported by the phylogenetic placement of the PpanHVLs in the tree shown in figure 4A, consistent with studies that have identified three distinct clades within the γ_1_ viruses that roughly correspond to major primate divergence events; new world monkeys, old world monkeys and hominoid hosts [32]. Figure 4B shows MaHV4 and PpanHVLs as a monophyletic group, which is inconsistent with a history of co-evolution. Interestingly, previous work has identified that MaHV4 and HHV4 are more closely related than expected, given the divergence date estimates for macaques and humans [19], [30]. In the figure 4A tree, the well-supported topology of the γ_1_ lineage reflects a topology more consistent with co-divergence. The clade containing all known old world monkey viruses also contains two separate lineages of hominoid hosts. This discordance is further complicated by the fact that the clade does not reflect their hosts' speciation pattern. The unusual grouping of hominoid viruses has been speculated to be the result of two cross-species transfers within the last 12 million years [33]. The first must have occurred from a monkey host to a hominoid, which either happened multiple times independently, or else resulted from a secondary transfer between hominoids. It is also thought that *Pongo pygmaeus LCV2* and *Symphalangus syndactylus LCV2* must have appeared in the last million years due to transmission from macaques to orang-utans and gibbons in Indonesia [33].

### PpanHVL genome characterisation

The bonobo sequences are composed of 9 contigs totalling 78,178 bp, and constitute approximately 45% of the expected total length of the virus, assuming a similar genome size to EBV. They contain ORFs that are homologous to 58 of the 94 EBV coding sequences, in precise co-linearity.

**Figure 5:**
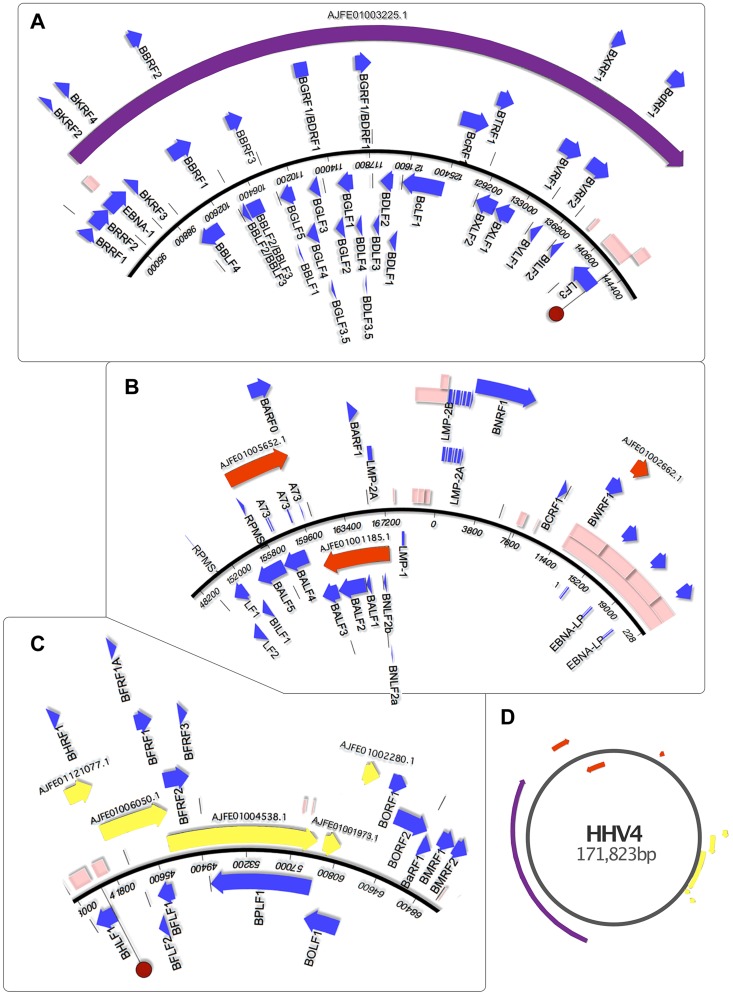
Genomic mapping of *Pan Paniscus* viral sequences. The viral sequences found in the *Pan paniscus* genome appear to represent parts of the *P. paniscus LCV1*, which had been previously been identified but only partially sequenced. Because of the extreme genetic similarity to *human herpesvirus 4* (HHV4) and identical gene set, ORF visualization was possible by aligning the contigs directly to the HHV4 genome. The contigs were separated into panels A–C to for clarity, and a zoomed out layout of the contigs is shown in panel D. In each panel, the blue-boxed arrows indicate the ORFs of HHV4, which are identically positioned in the PpanHVLs. Pink boxes represent repetitive sequence regions. In panel A and C, the dark red circle indicates the HHV4 origins of replication. It is interesting to note the repetitive sequences in regions of HHV4 that correspond to nearly all the edges of the PpanHVL contigs. Assembly algorithms are known to struggle in the reconstruction of low complexity sequences, which strongly suggests that for the *P. paniscus LCV1 genome*, repetitive sequences are located in the same place as they are in HHV4.

Figure 5A describes the correspondence between EBV and contig AJFE01003225.1, spanning 44,035 bp with 89% identity. It encodes several genes of the lytic cycle; the 5′ end begins with a partial coding sequence similar to EBNA1, the only gene that is expressed during both latent and lytic states. It is homologous to a region in EBV that is flanked by the short internal repeat regions IR3 and IR4, suggesting that AJFE01003225.1 terminates where it does because the PpanHVL virus also contains repetitive sequences at the same loci; genome assemblers struggle to consolidate short reads that span such low complexity regions.

EBNA1 is known to disrupt cellular antiviral/tumour suppressing ability and is expressed in association with several malignancies (reviewed in [5], [34]). Next, there are ORFs similar to the early lytic cycle genes BKRF2, BKRF3 and BKRF4, expression of which is controlled epigenetically by BZLF1 (absent in the PpanHVLs), which targets methylated CpG-containing binding sites [35]. The next ORFs are homologues of BBLF4, BBRF1, BBRF2, BBLF2/3 and BBRF3. BBLF2/3 and BBLF4 are known to interact with DNA polymerase (BALF5) and BSLF1 [36], as part of the lytic replication complex [37]. A PpanHVL BALF5 homologue is present (figure 5B), but BSLF1 is presumed to be in the missing regions. The next three ORFs (BBLF1, BGLF4 and BGLF5) are similar to a complex EBV region that is involved in lytic replication, and are transcribed into mRNAs with the same 3′ polyA signal [38]. The remainder of the contig encodes a number of genes that maintain precise synteny with EBV, and a homologue of BcRF1 is particularly interesting with respect to its disease association. BcRF1 is a captured host pleiotropic cytokine Il-10 that performs immunoevasive functions. It has been shown to reduce thymocyte enhancement and mast cell proliferation in mice, and can also cause a decrease of components in the antigen-processing pathway (reviewed in [39]).

Contig AJFE01005652.1 (figure 5B) contains homologues of glycoprotein B and DNA polymerase genes, which are also the genes with near identity to the only two previously described PpanLCV1 sequences. There is also similarity to BARF1, a secreted lytic protein involved in EBV immune evasion by inhibiting the release of Interferon-α from monocytes [40]. Contig AJFE01001185.1 exhibits similarity to 7 EBV genes, including latent membrane antigen LMP-2A and BNLF2a, which is an important inhibitor of the HLA class I antigen presentation pathway (unique to lymphocryptoviruses of old word primates [41]). EBV contains a block of internal repeats, part of which is detectible on contig AJFE01002662.1, which also encodes the last 254 aa of BWRF1.

The contigs depicted in figure 5C represent a 17,944 bp contiguous stretch containing 8 ORFs with exact correspondence to the equivalent EBV region. The first of these, BHRF1 is known to inhibit cell death by interacting with several apoptotic inducers [42]. Thereafter there is similarity to BFLF2 and BFLF1 that are involved in virion maturation [43], [44], and then homologues of BFLF1 and BFRF1A, involved in DNA packaging - their deletion results in genome-less virions, making them an ideal vaccine target since a CD4+ T-cell response is maintained [45]. The ORFs after these are similar to BFRF2, BFRF3 and BPLF1, which deubiquitinates (thereby disrupting) the proliferating cell nuclear antigen [46]. The last two contigs in figure 5C include similarity to BOLF1, a core herpesvirus tegument gene. The similarity to EBV is relatively low, with 70% identity over the 449 amino acids present in the PpanHVL contigs. A bulk of the difference between them lies in a 19 aa region that includes a 6 aa stretch that has been hypothesized to mimic self HLA-DPB1*0201, thereby being involved in T-cell reactivity to self in patients with juvenile idiopathic arthritis [47].

### DmadHVL genome characterisation

The DmadHVL contigs were particularly fragmented; the scaffolding data had not been released and the contig N50 was much smaller than the other two genomes (table 1). For this reason, we devised a targeted scaffolding strategy to link contigs that appeared close together based on the reference virus. This involved an iterative sequence-search for the edges of each contig, by reformatting the mate-pair reads into separate BLAST databases. The resulting assembly of mate pairs to the edge of their respective contigs allowed extension of the edge by tens of bases at a time. These new edges were then used to search for overlaps with contigs from the original wgs draft assembly that had previously not been identified by the original tBLASTn search. Scaffolding software and contig extension techniques do exist, but they are designed with the aim of improving the genome assembly as a whole. The brute-force approach employed here takes advantage of Illumina sequence reads, bridging the gaps between contigs in a sequence-specific manner. The labour-intensive dependence on ‘eye-ball’ quality assessment, means that it is inappropriate for large-scale contig extension, and would be difficult to automate fully. Indeed, it was often the case that multiple sets of read pairs were equally well aligned to the edge, and the decision process was aided by knowledge of the expected coding content. This means that the technique is particularly poor for contig edges that are non-coding. The sequence for the composite contigs can be found in supporting information S1.

**Figure 6:**
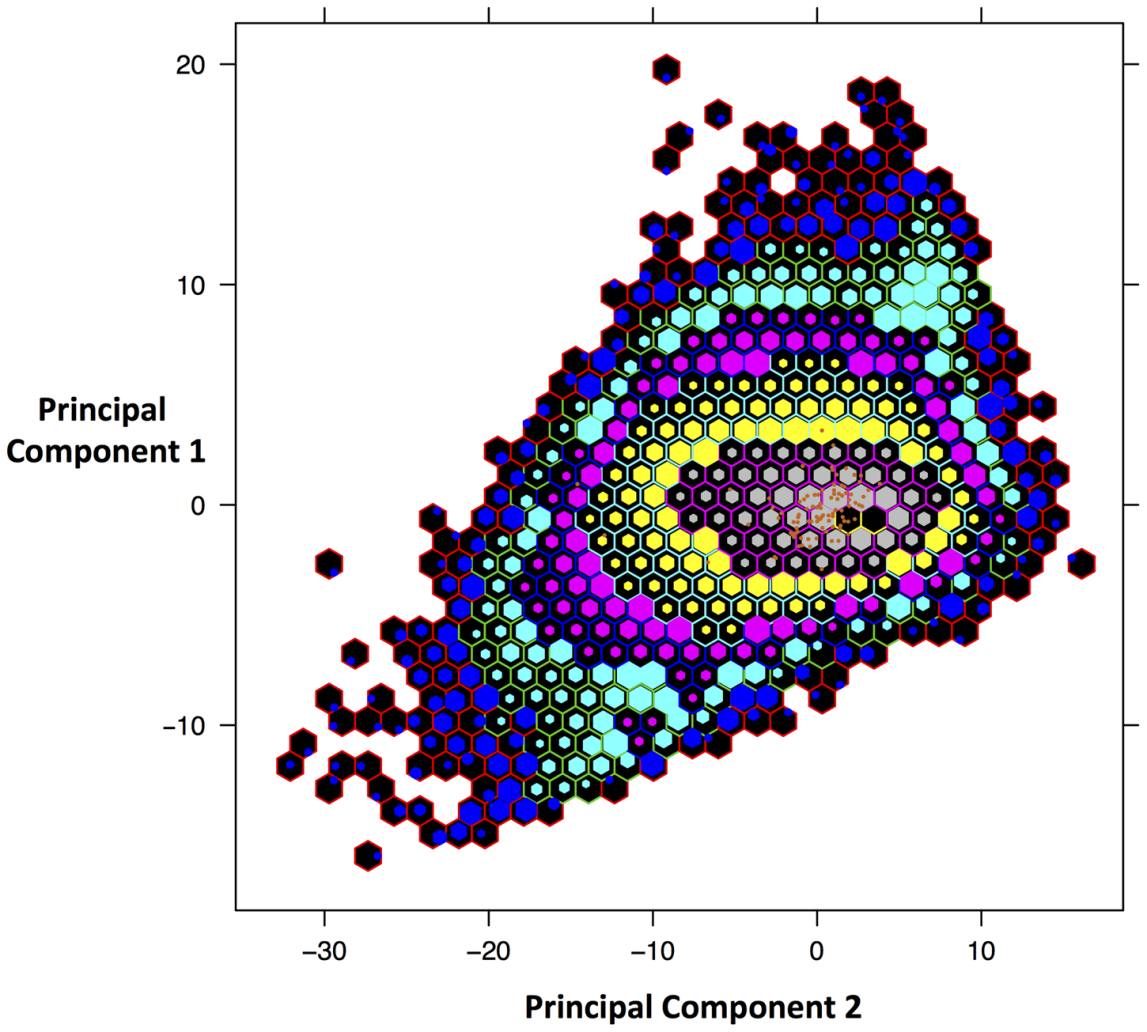
Principal Components Analysis of *D. madagascariensis* contigs. Plot of scores from the first 2 principal components that account for 77% of the variance. The variables are: read coverage depth, all four mononucleotide and sixteen dinucleotide frequencies, mean, median, minimum and maximum insert size of reads. Scores were binned in order to more easily view the distribution. Orange points represent the contigs identified as viral, which have been over-plotted to identify where in the distribution the lie. Each hexagon represents a different bin and the size of the internal hexagon represents how ‘full’ that bin is in terms of the number of contigs. The placement of the internal hexagon describes the mean value of scores in that bin. The number of contigs contained within each bin is represented by the following colour scheme: blue with red border: 1–9, turquoise with green border: 10–99, pink with blue border: 100–999, yellow with turquoise border: 1000–9999, grey with pink border: 10,000–99,999, Black with yellow border: 100,000–999,999.

The phylogenetic analyses and evidence from sequence similarity indicate that the DmadHVLs probably represent part of a previously undescribed rhadinovirus. As well as not detecting any nonsense mutations, we compared the nucleotide composition and mate-pair characteristics of viral contigs to the rest of the genome, in order to assess any differences that might better indicate they were part of an endogenous or exogenous virus. A principal components analysis of the assembled contigs was performed using 28 variables (figure 6). This revealed that the viral and non-viral contigs do not significantly differ, but it is nonetheless reassuring that the viral sequences cluster together, which is supportive of their shared identity. The overall genomic architecture of viruses in this group consists of a long unique region flanked by terminal repeat sequences, and the DmadHVLs adhere to this plan. Though it was not possible to determine the termini fully, regions in contig 1 and 16 both exhibit similarity to v-FGAM synthase, which is also found in other rhadinovirus genome extremities (figure 3C). In Contig 1, the 5′ region contains similarity to the complement control protein, which is involved in active immune evasion in the closely related human herpesvirus 8 (HHV8), the etiological agent of Kaposi's sarcoma [48]. The corresponding gene in SaHV-2 is situated between v-FGAM (ORF 3) and the single-stranded binding protein. Instead, the DmadHVLs possess an ORF at that position with similarity to bcl2, a family of cellular apoptosis regulators. Similar host-derived bcl2-like genes can be found in other rhadinoviruses, such as ORF16 in HHV8, and are known to either promote or inhibit apoptosis [49]. The DmadHVL version appears more similar to versions in *porcine lymphotropic herpesvirus* 1 and 2, *Ovine herpesvirus 2* and *Equine herpesvirus 2*, none of which are rhadinoviruses, albeit belonging to *Gammaherpesvirinae*. Another region of interest on contig 1 is ORF2, which shows similarity to BoHV4 pB05. The first three genes of the herpes core block 1 (HCB1) can be found in the DmadHVLs, but contig 1 ends before the end of glycoprotein B.

In every other rhadinovirus, the glycoprotein B and polymerase genes are invariably found in tandem and in the same coding direction. Contig 2 contains polymerase (ORF2) but is preceded by ORF1, which is a truncated homologue of glycoprotein H. It could be that this is due to erroneous assembly, or even a sequencing error, since we would expect glycoprotein H further downstream according to the genome plan of other rhadinoviruses. Indeed, we find that the first ORF in contig 5 shows similarity to glycoprotein H. Contig 3 shows similarity to the first two ORFs after HCB1 that are conserved in several other herpesviruses and likely derived from host-captured dUTPase, thereafter becoming internally duplicated in different lineages independently [50]. The 5′ end of contig 3 contains a short stretch of sequence similarity to Ov5, a G-protein coupled receptor found in the *Ovine herpesvirus 2*, *Alcelaphine herpesvirus 1*, and EBV BILF1, which share distant similarity to host interleukin 8 receptor [51]. The viral protein interferes with RNA-dependent protein kinase metabolism, which ordinarily acts to arrest cellular protein synthesis and induce apoptosis as an anti-viral defence. It was shown that the equivalent gene in SaHV2 was captured from the host [52]. Although the region is very short, it is unlikely to be a false positive, since it would be expected at that position given the colinearity with SaHV2.

In contig 4, ORF2 and 4 are similar to conserved core herpesvirus tegument protein and the protease/minor capsid protein, respectively. ORF 1 and 2 are also conserved in several herpesviruses, though their function has not been characterised. The third ORF of HCB2 falls in the data gap between contig 4 and 5. On contig 5, other than glycoprotein H, the subsequent 8 ORFs lie in a contiguous stretch of 13,440 bp. They are similar to the remaining HCB2 ORFs 23–26 as well as ORF27 and 28 and the first HCB3 ORFs 29–32. ORF32 is the DNA cleavage and packaging gene and is split between the two contigs 5 and 6, before the second exon of ORF 29. Alkaline exonuclease, a conserved herpesvirus gene superfamily straddles the gap between contig 6 and 7. The bulk of the gene is in contig 6 with 73% identity to SaHV2. Whereas its function in nearly all characterised genomes is involved in DNA replication and packaging, an additional function in HHV8 grants the ability to interfere at the mRNA level to shut off host gene expression [53]. Although none of the mutations implicated in HHV8's gain-of-function are shared by ORF1 contig 6, the host shut-off ability is attributed to structural differences, implying that it might not be limited to these specific positions/residues [54]. It would be interesting to know whether any such activity is present in the DmadHVL alkaline exonuclease.

Contig 8, 9 and 10 have the same HCB3 coding content and co-linearity with SaHV2, although the HCB3 ORF44 is entirely missing in the gap between 8 and 9. Interestingly, the first ORF on contig 9 exhibits higher similarity to the HHV8 ORF45, which blocks the activity of cellular interferon regulatory factor 7, an important part of cellular antiviral defence [55]. Contig 10 contains genes with similarity to the inter-HCB region, including the RTA transactivator that is involved in the switch from a latent to lytic infection state [56]. Whereas Contig11 does contain the first 6 genes of HCB4 as expected, there is a larger preceding region than in SaHV2. In the first ∼1.2 kb there is similarity to a gene of unknown function found in *porcine lymphotropic herpesvirus 1* (GenBank: AAM22152.1). The gap preceding contig 12 is presumed to contain HCB4 ORF57. Contig 12, 13 and 14 are 23,152 bp combined, and consist of separate genes; the DmadHVL ORF64 homologue is split over contig 12 and 13. Contig 14 begins with the last HCB4 ORF65–67, and then contains ORF67.5, a gene found in some rhadinoviruses but not SaHV2, the most similar of which is the Bovine herpesvirus 4 version. The contig ends with homologues of ORF68 and 69. Contig 15 contains similarity to ORF73, the latent nuclear antigen, a highly immunogenic product found in several other viruses (e.g. EBNA1 in EBV), but is most similar to the HHV8 version. LANA is essential for episomal genome maintenance [57], and is involved in a multitude of cellular interactions [58] and mechanisms of pathogenesis; it can upregulate cellular proliferation as well as prolong the cell cycle and inhibit apoptosis [57], [59]–[61].

## Discussion

Each of the HVLs sets were characterised with publically accessible data and open source software, demonstrating the utility of combining metagenomics and paleovirology to the study of viral diversity. Given the difficulty of sequencing herpesvirus genomes, our results highlight the value of mining genomic sequence databases in this way. They exemplify the advantage of paleovirological approaches to genome analysis that view nucleotide repositories as a single resource, containing more biodiversity than the sum of its contributing organisms. In the era of high throughput sequencing methods, all genomic data has the potential to be treated as metagenomic and it is important to highlight that the viruses described here were obtained not through technical innovations, but rather, by reframing the strategic approach and borrowing the paleovirological perspective.

The TsyrHVLs represent a Roseolovirus-like endogenous viral element, and originates from a previously undescribed species that we tentatively name *Tarsius syrichta roseolovirus 1* (TsRV1), or Tarsier herpesvirus EVE (THEVE) when referring to the paleovirus. THEVE clearly demonstrates the endogenisation of herpesviruses, and has extended the host range of roseoloviruses from being ape-exclusive to include tarsiers. This suggests that the lineage may be associated with many more primates; the only exogenous roseoloviruses identified to date infect chimpanzees [62] and humans. Previous studies have shown that the topology and relative branch lengths of betaherpesviruses are largely congruent with that of their hosts, which is strongly suggestive of co-divergence [3]. They are thought to have arisen by co-speciation with euarchontoglires hosts, but the lineage containing roseoloviruses deviates from this pattern and must have arisen by cross species transmission [3], [4]. There is not enough information to conclude whether single or multiple transmission events occurred, but if the lineage arose from a single spill over, then the identification of THEVE suggests that roseoloviruses have been infecting primates since the *Tarsiiformes* divergence approximately 81 mya [22], and our estimate for the integration date of THEVE between 55–75mya is consistent with this scenario.

Studies of ciHHV6 have confirmed that the telomeres are the chromosomal target for integration, and Marek's disease virus is also known to integrate into the telomere of chicken chromosomes, albeit not heritably (reviewed in [10]). As well as the presumed dependence of integration on homologous recombination of viral and host TMRs, the preference for telomeric regions is likely to have evolved because the large genomes of herpesviruses would not be tolerated elsewhere in the chromosome. Indications that HHV6 may integrate into some chromosomes preferentially suggest that even the telomeres of some chromosomes are too crucial to withstand such a drastic integration [15], [63]. Moreover, although the precise telomeric integration site is not specific in HHV6, it is unlikely to be evolutionarily stable at the extreme end of the telomere, which is gradually shortened at each cell division [64]. This raises the possibility that the region between the sub-telomere and chromosomal side of telomeres contains an underappreciated diversity of EVEs that would be very difficult to study. THEVE offers a way to study the long-term effects of ciHHV6 on both telomere biology and the general physiological and evolutionary impact of acquiring ∼150 kb of viral genomic material. It is also an alternative investigative avenue in which to fully characterise the *Roseolovirus* integration model. The mechanism of HHV6 integration has yet to be confirmed, and although a number of models have been proposed, it is difficult to assert with any confidence which models are applicable to THEVE [10], [11], [14], [63], [65]. There is strong evidence to suggest that the HHV6 capacity to integrate is brokered by its captured copy of *rep* [63]. It has been demonstrated that HHV6 can rescue replication of adeno-associated virus with defective *rep* [66]. We cannot know the exact process that lead to herpesvirus *rep* acquisition, but if the only selective advantage were conferring the ability to integrate, it would mean that the mechanism of *rep* transfer to roseoloviruses is also the reason for its evolutionary maintenance, and in the case of THEVE, the eventual genomic fossilization. Very little is known about how non-retroviral EVEs are formed, and in this case it would be interesting for future work to test the idea that the endogenisation of a parvovirus into a herpesvirus is in turn responsible for the endogenisation of the herpesvirus itself. Interestingly, although THEVE resolves basally to HHV6 and 7, the gene is absent in HHV7, which in fact has not been shown to be capable of genomic integration. A phylogeny of the gene comparing the copies of endogenous and exogenous parvoviruses robustly places the THEVE and HHV6 *rep* as sister taxa with 98% bootstrap support (figure S2), which is consistent with a common origin. We propose that the combined evidence of positional orthology and co-linearity of the *rep* gene in THEVE and HHV6, along with monophyly of the genes in the *rep* tree, suggest the subsequent loss of the gene from HHV7.

The data presented also includes the description of sequences that appear to be from exogenous viral contaminants of their host genome projects. In doing so, we have described a number of virulence genes shared between these viruses and pathogenic human herpesviruses. Our results indicate that the PanHVLs almost certainly represent the previously recognised *Pan paniscus lymphocryptovirus 1* (PpanLCV1), for which only two genes had been previously sequenced. The DmadHVL sequences are part of a previously undescribed rhadinovirus of aye-ayes, provisionally named *Daubentonia madagascariensis rhadinovirus 1* (DmRV1). For both viruses, the assembly status of the genomes meant that the viral sequences were composed of discontiguous stretches homologous to reference viruses. Interestingly, in the case of the largest PpanHVL contig that spanned over 40 kb, the homologous region of HHV4/EBV is flanked by internal repeat regions; a genome assembler would not have been able to reconstruct the junction easily (figure 5). Nonetheless, neither set of sequences contains disruptive mutations in coding sequences, and can be easily aligned to closely related and well-characterised herpesvirus genomes. The exogenous status of DmRV1 is further supported by the lack of any detectable viral sequences in the recently released re-sequencing project of 12 aye-aye individuals [67]. Both BLAST and read-mapping techniques were employed to search for HVL sequences in the other genomes, with no reportable matches. This also suggests that the virus is variably present in the population, and given that aye-ayes are a near-threatened species, understanding the factors that influence this will be informative from a conservation standpoint.

The techniques used to describe the exogenous viruses demonstrate the possibility of circumventing some of the challenges that clinical viral genomics is encumbered by. The relative paucity of viral nucleic acid compared to that of the host is a particular obstacle, and viral enrichment steps coupled with genetic variability risk introducing biases [68]. Doing this in the absence of host genome information is especially important due to the perpetual threat of emerging diseases by zoonosis [69], and the primate viruses we describe belong to lineages that include human pathogens (EBV, HHV6 and HHV7, HHV8). The herpesvirus capacity to cross species has been demonstrated experimentally [70], and such a phenomenon has already been observed in the case of the *Macacine herpesvirus 1*, an alphaherpesvirus of macaques that is deadly to humans [71]. The PpanLCV1 gene set is identical to that of EBV, meaning the overall pathology will likely be similar given that chimpanzees are physiologically comparable to humans. This makes it a substantial zoonotic risk, but also means PpanLCV1 an ideal system for comparative molecular analyses that may contribute to functional studies of EBV.

Across rhadinoviruses, the genomic regions that are most divergent (both genetically and in terms of gene-content) were also the regions that we could not recover for DmRV1. Namely, these are the terminal regions, as well as regions which are consistently gene-sparse in each species. These sequences are notoriously difficult to identify, needing dedicated treatment in genome characterisation, as was done, for example, in the recently described elephant endotheliotropic herpesviruses [72]. Nevertheless, the genomic arrangement closely resembles *Ateline herpesvirus 3* and *Saimiriine herpesvirus 2* (figure 3), and the vast majority of ORFs are characteristic of rhadinoviruses, which allows us to speculate on its likely pathogenic mechanisms. The presence of an ORF similar to the RTA gene tells us that like most herpesviruses, DmRV1 is capable of switching between latent and lytic replication, which means that infected animals are probably largely asymptomatic. Conversely, the LANA-like ORF on contig 15 indicates that the virus may be capable of upregulating cellular proliferation, potentially promoting tumorogenisis in certain circumstances. The *Rhadinovirus* lineage as a whole probably arose by cross species transmission [2], followed by multiple subsequent transmission events, raising the question of why rhadinoviruses are particularly amenable to host switching. It would be important for future work to address this, and DmRV1 will be key to understanding the origins and cross-species transmissibility of rhadinoviruses. Although the genome most closely resembles rhadinoviruses overall, we have described a number of similarities to other groups, such as the partial Ov5 gene and captured bcl2 homologue. Combined with the phylogenetic placement as the most basal member of the group this suggests that DmRV1 still maintains features of the transitional ancestor that probably began infecting primates in the late Cretaceous.

The techniques employed in this work were developed for the study of endogenous viral elements and have proved capable of identifying valuable viral genetic data embedded in online databases. Our objective was to address the lack of herpesviruses in the genomic fossil record, and so we incorporated metagenomic approaches to overcome bioinformatic hurdles such as their large genomes. In addition to describing the first herpesvirus EVE, the serendipitous identification of two probable exogenous viruses reveals a wider benefit of this multidisciplinary perspective. By establishing this proof-of-principle on both a paleovirus and accidentally sequenced viruses, there are promising practical applications in other areas of virology and general genomics that may have far reaching consequences.

## Materials and Methods

All computational analyses were conducted on an Apple Mac Pro running MAC OSX 10.7.5 with a 12-core Intel Xenon processor and 64GB RAM. Automation of tasks was achieved by a variety of custom scripts written in Python. The overall strategy involved searching for amino acid similarity in a 6-frame translation of a nucleotide sequence database [7], [73]. This is because a combination of high sequence divergence and frame-shifting mutations, often render the nucleotide sequences of EVEs undetectably dissimilar in a single-frame search. The ideal query protein represents the viral diversity well, but is simultaneously evolutionarily conserved enough to detect distant sequences. Poor sequence conservation across the order *Herpesvirales* means that there are no genes or domains that capture sequence diversity in this way, and to compensate by using a large query set would be computationally expensive. Instead, 33 separate conserved protein clusters from the *Alloherpesviridae* and *Herpesviridae* families were used to generate consensus sequences and used to query the NCBI databases WGS and RefSeq. BLAST alignments from aye-aye (*Daubentonia madagascariensis*), bonobo (*Pan paniscus*) and tarsier (*Tarsius syrichta*) indicated that similarity from multiple viral genes appeared syntenically on single contigs, motivating further investigation (table S1).

### Sequence collation

The genome records for the three primate species under investigation are at various levels of assembly completion (table 1). Consequently, the exercise of characterising viral sequences embedded within them is complicated by their fragmented state. Establishing a biological explanation for the presence of these HVLs requires an assessment of their identity, i.e., whether or not these are endogenous or merely a contaminant. Evidence that would suggest that these are EVEs includes finding the 5′ and/or 3′ flanking host genome regions, which would show that viral genes are integrated. Furthermore, the presence of mutations that result in frame shifts and stop codons in genes would indicate that the HVLs had been subject to random genetic drift as would be expected from EVEs. Herpesviruses are known to integrate chromosomally, however (reviewed in [10]), and some EVEs have been captured by the host genome to provide a function [24], [74], [75]. This means that the absence of inactivating mutations would not rule out endogenisation, nor would it necessarily be confirmed by their presence alone. Rather, a combination of different lines of evidence together builds a case for establishing the nature of these HVLs.

### Database mining

Locally implemented NCBI BLAST+ was used to run a tBLASTn search of the genomic databases wgs and RefSeq. The set of queries used were consensus sequences obtained from 33 different ‘protein clusters’ of *Herpesviridae* and *Alloherpesviridae*, which were conserved to at least the sub family level, and represented a broad functional range (table S3). We performed a qualitative assessment of the resulting alignments, in order to identify host species that might warrant further investigation. The bonobo, aye-aye and tarsier were shortlisted for the second search phase. The best matching viruses to contigs from each species belonged to the *Lymphocryptovirus*, *Rhadinovirus*, and *Roseolovirus* genera respectively, as determined by a reciprocal BLAST search of the contigs against a viral database of protein products. Delimiting the searchable database to only these species, allowed a more exhaustive search, using all protein sequences from the best viral groups. The resulting list of sequence records from each genome was then subjected to a final round of shortlisting. BLAST hits from each genome to viral proteins under 50 amino acids long or with an e-value score below 1E-10 were discarded from further investigation. Finally, a BLASTx search of these contigs against the NCBI protein database was performed to identify false positive hits, which can result from host homologues of viral proteins (table S1).

### Contig visualisation

The resulting set of BLAST hits were converted from XML to GenBank format using a custom Python script. These hits were then merged into the genome records of the viruses from which the protein queries were extracted, and used to generate a graphical representation of the host contigs using the reference viral genome as a guide. The map in figure 5 was created using a trial version of MacVector, while those in figure 1C, 2 and 3C were drawn using Pixelmator v2.2.2. Given the high sequence similarity to HHV4, it was possible to align the PpanHVLs to the reference genome (figure 5). As for the TsyrHVLs, it was not possible to predict the ORFs due to the abundance of stops, and their genetic distance to the reference roseoloviruses meant that an alignment would not be informative in terms of gene mapping. Instead, we used BLASTx results to evaluate the regions of similarity in the TsyrHVLs to known coding sequences.

### PCR amplification

DNA from *Carlito syrichta* (syn. *Tarsier syrichta*) was kindly provided by Dr. Christian Roos from the Gene Bank of Primates at the German Primate Centre in Göttingen, Germany. The sample was obtained from the muscle tissue of a specimen from Frankfurt Zoo. A first round of PCR amplification was performed using *T. syrichta* genomic DNA extracted from muscle. Two reactions with a final volume of 50 ul included 25 ul RedTaq Ready mix (sigma-aldrich), 1.5 ul 10 uM forward and reverse primer, 20 ul DNase/RNase free water, and two different concentrations of template. In one reaction, we used 2 ul of template at ∼15 ng/ul and 1 ul of ∼46 ng/ul in the other. Primers used were F4:GGCAGAAGATGGCACCTCC with R4:CCAACCCATTGAACTGCTAAAATTCC. Cycling parameters were: 2 minutes pre-denaturation at 94**°**C followed by 40 cycles of 40 seconds denaturation at 94**°**C, 40 second annealing at 55**°**C and 2 minute extension at 72**°**C, and a final extension at 72**°**C for 7 minutes. PCR purification was performed using QIAquick PCR purification kit by Qiagen, and although no bands were visible by agarose gel electrophoresis, DNA concentration of elution was performed using a nanodrop spectrophotometer indicating 6 and 8.3 ng/ul was present in the samples. In the second round of amplification, 2 ul of each of the first round amplicons were used in a reaction mix with the same ratio of reagents. Primer pairs used in each reaction were: F2:GGGATCACTGAATAAAGTGCGATGG, with R4:CCAACCCATTGAACTGCTAAAATTCC and F3:GGCATTTAAATTGACGAGTCCAAACGG with R4. Cycling parameters for second round were: 2 minutes pre-denaturation at 94**°**C followed by 20 cycles of 40 seconds denaturation at 94**°**C, 40 second annealing at 55**°**C and 2 minute extension at 72**°**C. We then ran a further 20 cycles with annealing temperature dropped to 50**°**C, and a final extension at 72**°**C for 7 minutes. Amplicons were visualised by 1% agarose gel electrophoresis (Figure 2A). Purified products were diluted to a concentration of 20 ng/ul and sent for Sanger sequencing by a commercial service (Source Bioscience Oxford). The sanger method cannot achieve good quality sequencing beyond ∼700 bp, so we used internal primers for the sequencing reaction as well as those used for amplification. The primers sequences mapped in figure 2 are: F27:GGTAAGTAGAGGTATAGCGGTAGTGTG, R27:CTTAGCCCATGTTTTACACCTTTAAG comp_R27:CTTAGCCCATGTTTTACACCTTTAAG, in_aluF:TAGGCATTGCCAAAAGAACA, R3:GGATTCAATGAAGGAGAATGG, R18:AACCTGGCTGCTAGCATCTG. The fragments sequenced from within the unique viral region were amplified using the cycling parameters as the first round of junction PCR, and the primers used on contig ABRT02259801.1 are F1:TGAGGGTTGCAGGTTGGTTT, R1:CCCGATATTCATCAGGCCCC; F2:TTCTGCAGTAAGGGCGAGTG, R2:TTCCTTGCTTCGGCATCCAT, F3:GATCTGCAACGGACAGGTGA, R3:AGCACACTGTCAAACCCACA, F4:CTTCCGTGTGAACTCCTGCT, R4:CTTTCAGCCACACAACTGCC, F5:GGCTTGGCCTCCTGATTTCT, R5:GCCCTTTAGCAGCTGTACCA. Primer pairs for fragments on contig ABRT02337366.1 are F6:CTAGCTGGGTGCACAGTCTC, R6:GACCCAAGAGAAGACGAGCC; F7:TTGGAATGCCATCTGTCGCT, R7:TTGGGAGGAAGCTGAACCAC. Sequence for all amplified fragments can be found in supporting information S1, and have been assigned the GenBank accession ID KJ410306.

### Alignment and phylogenetic reconstruction

Amino acid alignments were initially generated using the Mega 5.0 implementation of MUSCLE, and thereafter adjusted manually, including the removal of unalignable blocks. Tests for the best-fit evolutionary model were performed in Prottest 3.0, and chosen according to the Akaike information criterion. Initially, trees were estimated in PhyML, but comparison with RaxML indicated that the latter software found trees with higher likelihoods. The final phylogenies were estimated by maximum likelihood in RaxML and statistical support for the trees was calculated via 1000 nonparametric bootstrap replicates. The phylogenetic trees in figures 1A, 3A and 4C represent a 639 amino acid alignment of a conserved region in DNA polymerase for 144 *Herpesviridae* taxa (including the HVLs), and the best model was LG+G. To more accurately examine the relationships within each subfamily, taxonomic coverage was sacrificed in exchange for viruses for which a larger number of genes had already been sequenced. A 5,095 long concatenated amino acid alignment of DNA polymerase, glycoprotein B, helicase/primase, ssDNA binding protein, transport protein and major capsid was used to generate the gammaherpesvirus phylogeny of 18 taxa (figure 3B and 4B). Best models for each partition were LG+G+F but fixed frequency estimates for glycoprotein B and DNA polymerase. For the betaherpesvirus tree in figure 1B, we used a concatenated alignment for 16 taxa of terminase, large tegument, uracil-DNA glycosylase, kinase, capsid and helicase. The best model in each case was LG+G with empirical frequency estimates for terminase, large tegument, and helicase. Lastly, a phylogeny that exploited more gammaherpesvirus sequences from the database was also reconstructed. This tree did not comprise as much taxonomic coverage as for DNA polymerase (73 taxa), but using two genes in the alignment allowed for better-supported nodes than in the initial tree intended for quick taxonomic placement. In this case, we used a concatenation of Glycoprotein B and DNA polymerase genes in an 876 amino acid alignment with LG+G as the best model in both cases. The phylogeny of captured parvovirus *rep* genes (figure S2) in herpesviruses was reconstructed from a 281 amino acid alignment of the *rep* NS1 domain from 20 taxa, with the LG+G+F model.

## Supporting Information

Figure S1 Schematic diagram showing general herpesvirus genome layouts. Panel A depicts the genome maps of the 6 general herpesvirus layouts, A–F as described (and adapted from) in [4]. Boxes/Arrow boxes represent major repetitive regions. Types A, B, C and E depict genome layouts that are flanked repetitive regions, while layout D represents genomes where the major repeats are not at the termini. Type C and E also contain internal repeats - in C the number of internal reiterations is variable, as are the terminal reiterations in B. Type A represents genomes with direct terminal repeat regions. Type F represents genomes without terminal repeats. Panel B shows the layouts of various herpesvirus genomes, including the HVLs and those used as mapping references. Green boxes indicate repetitive regions and dotted lines indicate the missing regions of the HVLs, where only significant gaps are represented. HHV4, SaHV2 and HHV6 are considered types C, B and A, respectively, as are the PpanHVLs, DmadHVLs and TsyrHVLs.
(TIFF)

Figure S2 Phylogeny of captured Parvovirus *rep* gene in herpesviruses. ML tree of parvovirus NS1 domain including endogenous parvoviruses in mammals, captured genes in herpesviruses and type species from each genus of *Parvovirinae*. The NCBI accession number for the contig on which each parvovirus EVEs is located is show along side each species name. Only bootstrap values above 50 are shown at nodes. Given the positional orthology of *rep* in the TsyrHVLs and HHV6a, this tree is consistent with a common origin for the gene capture event. This suggests that HHV7 subsequently lost the gene as our phylogenetic analysis indicates that TsyrHVLs from diverged from other roseoloviruses before the HHV6/HHV7 split.
(TIFF)

Table S1 Accession numbers for the protein cluster database hosted by NCBI of related sequences used for initial BLAST search for herpesviruses in wgs database as of September 2012.
(PDF)

Table S2 Summary of BLAST report detailing initial hits to bonobo, aye-aye and tarsier using consensus sequences of 33 NCBI protein clusters.
(PDF)

Table S3 The spreadsheet contains a catalogue of reciprocal BLASTx results. Shortlisted viral contigs were checked against protein databases in order to eliminate false positive results from earlier rounds of BLAST searching. Information for each of the viruses investigated in this study is found on separate sheets. Sheet 1: DmadHVLs, sheet 2: PpanHVLs, sheet 3: TsyrHVLs. ‘Top Alternative Hit’ documents the best non-viral BLAST hit if the best hit is viral, or else shows the top viral hit if the best hit is host. The latter scenario occurs for a false positive hit.
(PDF)

Text S1 Text file containing scaffolded DmadHVL contigs and sequences generated by PCR experiments.
(TXT)

## References

[1] Lavergne A, de Thoisy B, Pouliquen J-F, Ruiz-García M, Lacoste V (2011) Partial molecular characterisation of New World non-human primate lymphocryptoviruses. Infect Genet Evol 11: 1782–1789 10.1016/j.meegid.2011.07.017 21827873

[2] McGeoch DJ, Gatherer D, Dolan A (2005) On phylogenetic relationships among major lineages of the Gammaherpesvirinae. J Gen Virol 86: 307–316 10.1099/vir.0.80588-0 15659749

[3] McGeoch DJ, Rixon FJ, Davison AJ (2006) Topics in herpesvirus genomics and evolution. Virus Res 117: 90–104 10.1016/j.virusres.2006.01.002 16490275

[4] McGeoch DJ, Davison AJ, Dolan A, Gatherer D, Sevilla-Reyes EE (2010) Molecular Evolution of the Herpesvirales. In: Domingo E, Holland JJ, editors. The Origin and Evolution of Viruses. Academic Press. 447–475 p. doi:10.1002/9780470688618.taw0208.

[5] Fields BN, Knipe DM, Howley PM (2007) Fields Virology. 5th ed. Philadelphia: Lippincott Williams and Wilkins.

[6] Tischer BK, Osterrieder N (2010) Herpesviruses–a zoonotic threat? Vet Microbiol 140: 266–270 10.1016/j.vetmic.2009.06.020 19616388 PMC2815145

[7] Katzourakis A, Gifford RJ (2010) Endogenous viral elements in animal genomes. PLoS Genet 6: e1001191. 21124940 10.1371/journal.pgen.1001191 PMC2987831

[8] Feschotte C, Gilbert C (2012) Endogenous viruses: insights into viral evolution and impact on host biology. Nat Rev Genet 13: 283–296 10.1038/nrg3199 22421730

[9] Hurley EA, Agger S, McNeil JA, Lawrence JB, Calendar A, et al (1991) When Epstein-Barr virus persistently infects B-cell lines, it frequently integrates. J Virol 65: 1245–1254. 1847452 10.1128/jvi.65.3.1245-1254.1991 PMC239896

[10] Morissette G, Flamand L (2010) Herpesviruses and chromosomal integration. J Virol 84: 12100–12109 10.1128/JVI.01169-10 20844040 PMC2976420

[11] Kaufer BB, Jarosinski KW, Osterrieder N (2011) Herpesvirus telomeric repeats facilitate genomic integration into host telomeres and mobilization of viral DNA during reactivation. J Exp Med 208: 605–615 10.1084/jem.20101402 21383055 PMC3058580

[12] Delecluse HJ, Hammerschmidt W (1993) Status of Marek's disease virus in established lymphoma cell lines: herpesvirus integration is common. J Virol 67: 82–92. 8380099 10.1128/jvi.67.1.82-92.1993 PMC237340

[13] Delecluse HJ, Schüller S, Hammerschmidt W (1993) Latent Marek's disease virus can be activated from its chromosomally integrated state in herpesvirus-transformed lymphoma cells. EMBO J 12: 3277–3286. 8393785 10.1002/j.1460-2075.1993.tb05997.x PMC413595

[14] Arbuckle JH, Pantry SN, Medveczky MM, Prichett J, Loomis KS, et al (2013) Mapping the telomere integrated genome of human herpesvirus 6A and 6B. Virology 10.1016/j.virol.2013.03.030 PMC3696530 23648233

[15] Arbuckle JH, Medveczky MM, Luka J, Hadley SH, Luegmayr A, et al (2010) The latent human herpesvirus-6A genome specifically integrates in telomeres of human chromosomes in vivo and in vitro. PNAS 107: 5563–5568 10.1073/pnas.0913586107 20212114 PMC2851814

[16] Pellett PE, Ablashi DV, Ambros PF, Agut H, Caserta MT, et al (2012) Chromosomally integrated human herpesvirus 6: questions and answers. Rev Med Virol 22: 144–155 10.1002/rmv.715 22052666 PMC3498727

[17] Delwart E (2013) A Roadmap to the Human Virome. PLoS Pathog 9: e1003146 10.1371/journal.ppat.1003146 23457428 PMC3573120

[18] Wilkie GS, Davison AJ, Watson M, Kerr K, Sanderson S, et al (2013) Complete Genome Sequences of Elephant Endotheliotropic Herpesviruses 1A and 1B Determined Directly from Fatal Cases. J Virol 87: 6700–6712 10.1128/JVI.00655-13 23552421 PMC3676107

[19] Ehlers B, Ochs A, Leendertz F, Goltz M, Boesch C, et al (2003) Novel simian homologues of Epstein-Barr virus. J Virol 77: 10695–10699. 12970457 10.1128/JVI.77.19.10695-10699.2003 PMC228477

[20] Kumar S, Subramanian S (2002) Mutation rates in mammalian genomes. PNAS 99: 803–808 10.1073/pnas.022629899 11792858 PMC117386

[21] Pace JK, Gilbert C, Clark MS, Feschotte C (2008) Repeated horizontal transfer of a DNA transposon in mammals and other tetrapods. PNAS 105: 17023–17028 10.1073/pnas.0806548105 18936483 PMC2579371

[22] Perelman P, Johnson WE, Roos C, Seuánez HN, Horvath JE, et al (2011) A molecular phylogeny of living primates. PLoS Genet 7: e1001342 10.1371/journal.pgen.1001342 21436896 PMC3060065

[23] Holmes EC (2011) The Evolution of Endogenous Viral Elements. Cell Host Microbe 10: 368–377 10.1016/j.chom.2011.09.002 22018237 PMC7172163

[24] Aswad A, Katzourakis A (2012) Paleovirology and virally derived immunity. Trends Ecol Evol 27: 627–636 10.1016/j.tree.2012.07.007 22901901

[25] Tadagaki K, Nakano K, Yamanishi K (2005) Human herpesvirus 7 open reading frames U12 and U51 encode functional beta-chemokine receptors. J Virol 79: 7068–7076 10.1128/JVI.79.11.7068-7076.2005 15890946 PMC1112110

[26] Thomson BJ, Efstathiou S, Honess RW (1991) Acquisition of the human adeno-associated virus type-2 rep gene by human herpesvirus type-6. Nature 351: 78–80 10.1038/351078a0 1851252

[27] McGeoch DJ, Dolan A, Ralph AC (2000) Toward a Comprehensive Phylogeny for Mammalian and Avian Herpesviruses. J Virol 74: 10401–10406 10.1128/JVI.74.22.10401-10406.2000 11044084 PMC110914

[28] Ehlers B, Dural G, Yasmum N, Lembo T, de Thoisy B, et al (2008) Novel mammalian herpesviruses and lineages within the Gammaherpesvirinae: cospeciation and interspecies transfer. J Virol 82: 3509–3516 10.1128/JVI.02646-07 18216123 PMC2268488

[29] McGeoch DJ (2001) Molecular evolution of the gamma-Herpesvirinae. Philos Trans R Soc Lond B Biol Sci 356: 421–435 10.1098/rstb.2000.0775 11313003 PMC1088436

[30] Gerner CS, Dolan A, McGeoch DJ (2004) Phylogenetic relationships in the Lymphocryptovirus genus of the Gammaherpesvirinae. Virus Res 99: 187–192 10.1016/j.virusres.2003.10.011 14749184

[31] Davison AJ, Eberle R, Ehlers B, Hayward GS, McGeoch DJ, et al (2009) The order Herpesvirales. Arch Virol 154: 171–177 10.1007/s00705-008-0278-4 19066710 PMC3552636

[32] Ehlers B, Spiess K, Leendertz F, Peeters M, Boesch C, et al (2010) Lymphocryptovirus phylogeny and the origins of Epstein-Barr virus. J Gen Virol 91: 630–642 10.1099/vir.0.017251-0 19923263

[33] Ehlers B, Spiess K, Leendertz F, Peeters M, Boesch C, et al (2010) Lymphocryptovirus phylogeny and the origins of Epstein-Barr virus. J Gen Virol 91: 630–642 10.1099/vir.0.017251-0 19923263

[34] Crawford DH (2001) Biology and disease associations of Epstein-Barr virus. Philos Trans R Soc Lond B Biol Sci 356: 461–473 10.1098/rstb.2000.0783 11313005 PMC1088438

[35] Ramasubramanyan S, Kanhere A, Osborn K, Flower K, Jenner RG, et al (2012) Genome-wide analyses of Zta binding to the Epstein-Barr virus genome reveals interactions in both early and late lytic cycles and an epigenetic switch leading to an altered binding profile. J Virol 86: 12494–12502 10.1128/JVI.01705-12 23015699 PMC3497672

[36] Fujii K, Yokoyama N, Kiyono T, Kuzushima K, Homma M, et al (2000) The Epstein-Barr Virus Pol Catalytic Subunit Physically Interacts with the BBLF4-BSLF1-BBLF2/3 Complex. J Virol 74: 2550–2557 10.1128/JVI.74.6.2550-2557.2000 10684269 PMC111743

[37] El-Guindy A, Ghiassi-Nejad M, Golden S, Delecluse H-J, Miller G (2013) Essential role of Rta in lytic DNA replication of Epstein-Barr virus. J Virol 87: 208–223 10.1128/JVI.01995-12 23077295 PMC3536415

[38] Chiu Y-F, Sugden B, Chang P-J, Chen L-W, Lin Y-J, et al (2012) Characterization and intracellular trafficking of Epstein-Barr virus BBLF1, a protein involved in virion maturation. J Virol 86: 9647–9655 10.1128/JVI.01126-12 22740416 PMC3446546

[39] Ressing ME, Horst D, Griffin BD, Tellam J, Zuo J, et al (2008) Epstein-Barr virus evasion of CD8(+) and CD4(+) T cell immunity via concerted actions of multiple gene products. Semin Cancer Biol 18: 397–408 10.1016/j.semcancer.2008.10.008 18977445

[40] Cohen JI, Lekstrom K (1999) Epstein-Barr virus BARF1 protein is dispensable for B-cell transformation and inhibits alpha interferon secretion from mononuclear cells. J Virol 73: 7627–7632. 10438853 10.1128/jvi.73.9.7627-7632.1999 PMC104290

[41] Hislop AD, Ressing ME, van Leeuwen D, Pudney VA, Horst D, et al (2007) A CD8+ T cell immune evasion protein specific to Epstein-Barr virus and its close relatives in Old World primates. J Exp Med 204: 1863–1873 10.1084/jem.20070256 17620360 PMC2118677

[42] Kvansakul M, Wei AH, Fletcher JI, Willis SN, Chen L, et al (2010) Structural basis for apoptosis inhibition by Epstein-Barr virus BHRF1. PLoS Pathog 6: e1001236 10.1371/journal.ppat.1001236 21203485 PMC3009601

[43] Lake CM, Hutt-Fletcher LM (2004) The Epstein-Barr virus BFRF1 and BFLF2 proteins interact and coexpression alters their cellular localization. Virology 320: 99–106 10.1016/j.virol.2003.11.018 15003866

[44] Gonnella R, Farina A, Santarelli R, Raffa S, Feederle R, et al (2005) Characterization and intracellular localization of the Epstein-Barr virus protein BFLF2: interactions with BFRF1 and with the nuclear lamina. J Virol 79: 3713–3727 10.1128/JVI.79.6.3713-3727.2005 15731265 PMC1075684

[45] Pavlova S, Feederle R, Gärtner K, Fuchs W, Granzow H, et al (2013) An Epstein-Barr virus mutant produces immunogenic defective particles devoid of viral DNA. J Virol 87: 2011–2022 10.1128/JVI.02533-12 23236073 PMC3571473

[46] Whitehurst CB, Vaziri C, Shackelford J, Pagano JS (2012) Epstein-Barr virus BPLF1 deubiquitinates PCNA and attenuates polymerase η recruitment to DNA damage sites. J Virol 86: 8097–8106 10.1128/JVI.00588-12 22623772 PMC3421674

[47] Massa M, Mazzoli F, Pignatti P, De Benedetti F, Passalia M, et al (2002) Proinflammatory responses to self HLA epitopes are triggered by molecular mimicry to Epstein-Barr virus proteins in oligoarticular juvenile idiopathic arthritis. Arthritis Rheum 46: 2721–2729 10.1002/art.10564 12384932

[48] Rezaee SAR, Cunningham C, Davison AJ, Blackbourn DJ (2006) Kaposi's sarcoma-associated herpesvirus immune modulation: an overview. J Gen Virol 87: 1781–1804 10.1099/vir.0.81919-0 16760382

[49] Kelly GL, Strasser A (2011) The essential role of evasion from cell death in cancer. Adv Cancer Res 111: 39–96 10.1016/B978-0-12-385524-4.00002-7 21704830 PMC3128425

[50] Davison AJ, Stow ND (2005) New genes from old: redeployment of dUTPase by herpesviruses. J Virol 79: 12880–12892 10.1128/JVI.79.20.12880-12892.2005 16188990 PMC1235826

[51] Hart J, Ackermann M, Jayawardane G, Russell G, Haig DM, et al (2007) Complete sequence and analysis of the ovine herpesvirus 2 genome. J Gen Virol 88: 28–39 10.1099/vir.0.82284-0 17170433

[52] Ahuja SK, Murphy PM (1993) Molecular piracy of mammalian interleukin-8 receptor type B by herpesvirus saimiri. J Biol Chem 268: 20691–20694. 8407886

[53] Glaunsinger B, Ganem D (2004) Lytic KSHV Infection Inhibits Host Gene Expression by Accelerating Global mRNA Turnover. Mol Cell 13: 713–723 10.1016/S1097-2765(04)00091-7 15023341

[54] Glaunsinger B, Chavez L, Ganem D (2005) The Exonuclease and Host Shutoff Functions of the SOX Protein of Kaposi's Sarcoma-Associated Herpesvirus Are Genetically Separable. J Virol 79: 7396–7401. 15919895 10.1128/JVI.79.12.7396-7401.2005 PMC1143623

[55] Zhu FX, King SM, Smith EJ, Levy DE, Yuan Y (2002) A Kaposi's sarcoma-associated herpesviral protein inhibits virus-mediated induction of type I interferon by blocking IRF-7 phosphorylation and nuclear accumulation. PNAS 99: 5573–5578 10.1073/pnas.082420599 11943871 PMC122811

[56] Damania B, Jeong JH, Bowser BS, DeWire SM, Staudt MR, et al (2004) Comparison of the Rta/Orf50 Transactivator Proteins of Gamma-2-Herpesviruses. J Virol 78: 5491–5499 10.1128/JVI.78.10.5491-5499.2004 15113928 PMC400334

[57] De León Vázquez E, Kaye KM (2011) The internal Kaposi's sarcoma-associated herpesvirus LANA regions exert a critical role on episome persistence. J Virol 85: 7622–7633 10.1128/JVI.00304-11 21593163 PMC3147901

[58] Kaul R, Verma SC, Robertson ES (2007) Protein complexes associated with the Kaposi's sarcoma-associated herpesvirus-encoded LANA. Virology 364: 317–329 10.1016/j.virol.2007.03.010 17434559 PMC4067005

[59] Watanabe T, Sugaya M, Atkins AM, Aquilino EA, Yang A, et al (2003) Kaposi's sarcoma-associated herpesvirus latency-associated nuclear antigen prolongs the life span of primary human umbilical vein endothelial cells. J Virol 77: 6188–6196. 12743275 10.1128/JVI.77.11.6188-6196.2003 PMC155023

[60] Fujimuro M, Hayward SD (2003) The latency-associated nuclear antigen of Kaposi's sarcoma-associated herpesvirus manipulates the activity of glycogen synthase kinase-3beta. J Virol 77: 8019–8030. 12829841 10.1128/JVI.77.14.8019-8030.2003 PMC161926

[61] Cai Q, Xiao B, Si H, Cervini A, Gao J, et al (2012) Kaposi's sarcoma herpesvirus upregulates Aurora A expression to promote p53 phosphorylation and ubiquitylation. PLoS Pathog 8: e1002566 10.1371/journal.ppat.1002566 22396649 PMC3291660

[62] Lacoste V, Verschoor EJ, Nerrienet E, Gessain A (2005) A novel homologue of Human herpesvirus 6 in chimpanzees. J Gen Virol 86: 2135–2140 10.1099/vir.0.81034-0 16033960

[63] Arbuckle JH, Medveczky PG (2011) The molecular biology of human herpesvirus-6 latency and telomere integration. Microbes Infect 13: 731–741. 21458587 10.1016/j.micinf.2011.03.006 PMC3130849

[64] Riethman H (2008) Human telomere structure and biology. Annu Rev Genomics Hum Genet 9: 1–19 10.1146/annurev.genom.8.021506.172017 18466090

[65] Huang Y, Hidalgo-Bravo A, Zhang E, Cotton VE, Mendez-Bermudez A, et al (2013) Human telomeres that carry an integrated copy of human herpesvirus 6 are often short and unstable, facilitating release of the viral genome from the chromosome. Nucleic Acids Res gkt840 10.1093/nar/gkt840 PMC3874159 24057213

[66] Thomson BJ, Weindler FW, Gray D, Schwaab V, Heilbronn R (1994) Human Herpesvirus 6 (HHV-6) Is a Helper Virus for Adeno-Associated Virus Type 2 (AAV-2) and the AAV-2 rep Gene Homologue in HHV-6 Can Mediate AAV-2 DNA Replication and Regulate Gene Expression. Virology 204: 304–311. 8091661 10.1006/viro.1994.1535

[67] Perry GH, Louis EE, Ratan A, Bedoya-Reina OC, Burhans RC, et al (2013) Aye-aye population genomic analyses highlight an important center of endemism in northern Madagascar. PNAS 110: 5823–5828 10.1073/pnas.1211990110 23530231 PMC3625347

[68] Renzette N, Bhattacharjee B, Jensen JD, Gibson L, Kowalik TF (2011) Extensive genome-wide variability of human cytomegalovirus in congenitally infected infants. PLoS Pathog 7: e1001344 10.1371/journal.ppat.1001344 21625576 PMC3098220

[69] Christou L (2011) The global burden of bacterial and viral zoonotic infections. Clin Microbiol Infect 17: 326–330 10.1111/j.1469-0691.2010.03441.x 21129102 PMC7129620

[70] Chang H, Wachtman LM, Pearson CB, Lee J-S, Lee H-R, et al (2009) Non-human primate model of Kaposi's sarcoma-associated herpesvirus infection. PLoS Pathog 5: e1000606 10.1371/journal.ppat.1000606 19798430 PMC2745662

[71] Huff JL, Barry PA (2003) B-virus (Cercopithecine herpesvirus 1) infection in humans and macaques: potential for zoonotic disease. Emerg Infect Dis 9: 246–250 10.3201/eid0902.020272 12603998 PMC2901951

[72] Wilkie GS, Davison AJ, Watson M, Kerr K, Sanderson S, et al (2013) Complete genome sequences of elephant endotheliotropic herpesviruses 1A and 1B determined directly from fatal cases. J Virol 87: 6700–6712 10.1128/JVI.00655-13 23552421 PMC3676107

[73] Horie M, Honda T, Suzuki Y, Kobayashi Y, Daito T, et al (2010) Endogenous non-retroviral RNA virus elements in mammalian genomes. Nature 463: 84–87 10.1038/nature08695 20054395 PMC2818285

[74] Belyi VA, Levine AJ, Skalka AM (2010) Unexpected inheritance: multiple integrations of ancient bornavirus and ebolavirus/marburgvirus sequences in vertebrate genomes. PLoS Pathog 6: e1001030 10.1371/journal.ppat.1001030 20686665 PMC2912400

[75] Cornelis G, Heidmann O, Bernard-Stoecklin S, Reynaud K, Véron G, et al (2012) Ancestral capture of syncytin-Car1, a fusogenic endogenous retroviral envelope gene involved in placentation and conserved in Carnivora. PNAS 109: E432–41 10.1073/pnas.1115346109 22308384 PMC3289388

[76] Prüfer K, Munch K, Hellmann I, Akagi K, Miller JR, et al (2012) The bonobo genome compared with the chimpanzee and human genomes. Nature 486: 527–531 10.1038/nature11128 22722832 PMC3498939

[77] Perry GH, Reeves D, Melsted P, Ratan A, Miller W, et al (2012) A genome sequence resource for the aye-aye (Daubentonia madagascariensis), a nocturnal lemur from Madagascar. Genome Biol Evol 4: 126–135 10.1093/gbe/evr132 22155688 PMC3273163

