# Supplementary figures and images for: The First Endogenous Herpesvirus, Identified in the Tarsier Genome, and Novel Sequences from Primate Rhadinoviruses and Lymphocryptoviruses
Source: PLoS Genet. 2014 Jun 19;10(6):e1004332. doi: 10.1371/journal.pgen.1004332 (PMC4063692; doi:10.1371/journal.pgen.1004332)

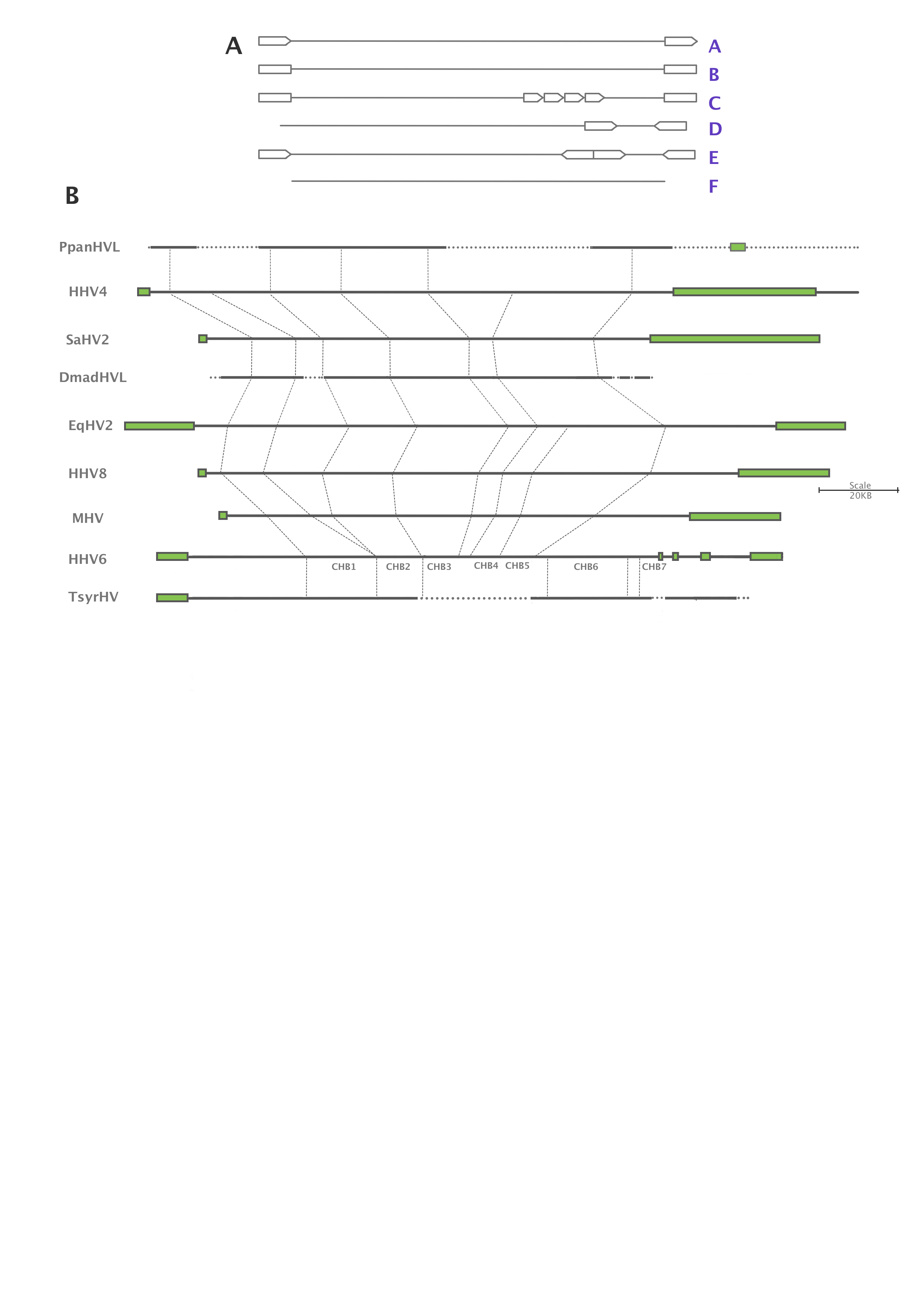

Supplement: Figure S1 — Schematic diagram showing general herpesvirus genome layouts. Panel A depicts the genome maps of the 6 general herpesvirus layouts, A–F as described (and adapted from) in [4]. Boxes/Arrow boxes represent major repetitive regions. Types A, B, C and E depict genome layouts that are flanked repetitive regions, while layout D represents genomes where the major repeats are not at the termini. Type C and E also contain internal repeats - in C the number of internal reiterations is variable, as are the terminal reiterations in B. Type A represents genomes with direct terminal repeat regions. Type F represents genomes without terminal repeats. Panel B shows the layouts of various herpesvirus genomes, including the HVLs and those used as mapping references. Green boxes indicate repetitive regions and dotted lines indicate the missing regions of the HVLs, where only significant gaps are represented. HHV4, SaHV2 and HHV6 are considered types C, B and A, respectively, as are the PpanHVLs, DmadHVLs and TsyrHVLs. (TIFF) [file pgen.1004332.s001.tiff]
