# Supplementary material for: The First Endogenous Herpesvirus, Identified in the Tarsier Genome, and Novel Sequences from Primate Rhadinoviruses and Lymphocryptoviruses
Source: PLoS Genet. 2014 Jun 19;10(6):e1004332. doi: 10.1371/journal.pgen.1004332 (PMC4063692; doi:10.1371/journal.pgen.1004332)
Supplement: Table S2 — Summary of BLAST report detailing initial hits to bonobo, aye-aye and tarsier using consensus sequences of 33 NCBI protein clusters. (PDF) [file pgen.1004332.s004.pdf]

Query	Subject taxon	Subject accession	Query start	Query end	Subject Start	Subject End	E-value	Identities
ATPase subunit terminase	Daubentonia madagascariensis	gi|368812353|gb|AGTM011948974.1|	331	540	1039	1641	0.000408279	50
hypothetical protein	Daubentonia madagascariensis	gi|370376862|gb|AGTM010395004.1|	77	256	690	1217	4.87E-24	65
hypothetical protein	Daubentonia madagascariensis	gi|368406985|gb|AGTM012354265.1|	250	461	4	606	8.92E-24	68
hypothetical protein	Daubentonia madagascariensis	gi|370222609|gb|AGTM010547017.1|	515	701	67	621	1.14E-86	139
hypothetical protein	Daubentonia madagascariensis	gi|370370516|gb|AGTM010401350.1|	694	813	1	354	1.48E-42	82
hypothetical protein	Daubentonia madagascariensis	gi|370745063|gb|AGTM010130173.1|	234	468	13	567	1.16E-14	71
hypothetical protein	Daubentonia madagascariensis	gi|370377335|gb|AGTM010394531.1|	40	213	1560	1985	3.97E-12	61
uracil DNA glycosylase	Daubentonia madagascariensis	gi|369057494|gb|AGTM011703833.1|	7	261	557	1309	1.73E-92	163
capsid triplex subutnit 1	Daubentonia madagascariensis	gi|370047245|gb|AGTM010722312.1|	15	231	102	671	5.57E-49	106
capsid triplex subutnit 1	Daubentonia madagascariensis	gi|370376421|gb|AGTM010395445.1|	268	383	41	385	3.43E-17	50
capsid triplex subunit 2	Daubentonia madagascariensis	gi|367791523|gb|AGTM012969727.1|	117	303	2	556	7.30E-47	114
capsid triplex subunit 2	Daubentonia madagascariensis	gi|370377301|gb|AGTM010394565.1|	1	120	2	361	4.85E-35	72
FGAM-synthase	Daubentonia madagascariensis	gi|368941814|gb|AGTM011819513.1|	481	1006	3	1406	1.14E-50	173
FGAM-synthase	Daubentonia madagascariensis	gi|368447111|gb|AGTM012314139.1|	1070	1529	630	1901	5.61E-49	154
FGAM-synthase	Daubentonia madagascariensis	gi|368989409|gb|AGTM011771918.1|	1093	1438	1	945	4.47E-38	124
DNA packaging terminase subutnit 1	Daubentonia madagascariensis	gi|368812353|gb|AGTM011948974.1|	448	791	1033	2025	2.94E-67	133
DNA packaging terminase subutnit 1	Daubentonia madagascariensis	gi|370377158|gb|AGTM010394708.1|	304	447	110	541	1.95E-15	48
DNA packaging prot UL32	Daubentonia madagascariensis	gi|370377307|gb|AGTM010394559.1|	484	716	367	942	2.81E-08	61
DNA packaging prot UL32	Daubentonia madagascariensis	gi|367815637|gb|AGTM012945613.1|	228	317	1060	1335	0.0871759	32
DNA packaging terminase subutnit 1	Daubentonia madagascariensis	gi|368812353|gb|AGTM011948974.1|	301	679	1033	2163	2.73E-89	166
DNA packaging terminase subutnit 1	Daubentonia madagascariensis	gi|370377158|gb|AGTM010394708.1|	126	300	35	541	1.80E-23	62
ATPase subunit terminase	Daubentonia madagascariensis	gi|368812353|gb|AGTM011948974.1|	331	540	1039	1641	0.000408279	50
hypothetical protein	Daubentonia madagascariensis	gi|370376862|gb|AGTM010395004.1|	77	256	690	1217	4.87E-24	65
hypothetical protein	Daubentonia madagascariensis	gi|368406985|gb|AGTM012354265.1|	250	461	4	606	8.92E-24	68
hypothetical protein	Daubentonia madagascariensis	gi|370222609|gb|AGTM010547017.1|	515	701	67	621	1.14E-86	139
hypothetical protein	Daubentonia madagascariensis	gi|370370516|gb|AGTM010401350.1|	694	813	1	354	1.48E-42	82
hypothetical protein	Daubentonia madagascariensis	gi|370745063|gb|AGTM010130173.1|	234	468	13	567	1.16E-14	71
hypothetical protein	Daubentonia madagascariensis	gi|370377335|gb|AGTM010394531.1|	40	213	1560	1985	3.97E-12	61
uracil DNA glycosylase	Daubentonia madagascariensis	gi|369057494|gb|AGTM011703833.1|	7	261	557	1309	1.73E-92	163
capsid triplex subutnit 1	Daubentonia madagascariensis	gi|370047245|gb|AGTM010722312.1|	15	231	102	671	5.57E-49	106
capsid triplex subutnit 1	Daubentonia madagascariensis	gi|370376421|gb|AGTM010395445.1|	268	383	41	385	3.43E-17	50
capsid triplex subunit 2	Daubentonia madagascariensis	gi|367791523|gb|AGTM012969727.1|	117	303	2	556	7.30E-47	114
capsid triplex subunit 2	Daubentonia madagascariensis	gi|370377301|gb|AGTM010394565.1|	1	120	2	361	4.85E-35	72
FGAM-synthase	Daubentonia madagascariensis	gi|368941814|gb|AGTM011819513.1|	481	1006	3	1406	1.14E-50	173
FGAM-synthase	Daubentonia madagascariensis	gi|368447111|gb|AGTM012314139.1|	1070	1529	630	1901	5.61E-49	154
FGAM-synthase	Daubentonia madagascariensis	gi|368989409|gb|AGTM011771918.1|	1093	1438	1	945	4.47E-38	124
DNA packaging terminase subutnit 1	Daubentonia madagascariensis	gi|368812353|gb|AGTM011948974.1|	448	791	1033	2025	2.94E-67	133
DNA packaging terminase subutnit 1	Daubentonia madagascariensis	gi|370377158|gb|AGTM010394708.1|	304	447	110	541	1.95E-15	48
DNA packaging prot UL32	Daubentonia madagascariensis	gi|370377307|gb|AGTM010394559.1|	484	716	367	942	2.81E-08	61
DNA packaging prot UL32	Daubentonia madagascariensis	gi|367815637|gb|AGTM012945613.1|	228	317	1060	1335	0.0871759	32
DNA packaging terminase subutnit 1	Daubentonia madagascariensis	gi|368812353|gb|AGTM011948974.1|	301	679	1033	2163	2.73E-89	166
DNA packaging terminase subutnit 1	Daubentonia madagascariensis	gi|370377158|gb|AGTM010394708.1|	126	300	35	541	1.80E-23	62
ATPase subunit terminase	Pan paniscus	gi|383075026|gb|AJFE01003225.1|	331	467	20383	20784	9.05E-05	43
hypothetical protein	Pan paniscus	gi|383075026|gb|AJFE01003225.1|	40	893	28569	30845	2.60E-157	372
uracil DNA glycosylase	Pan paniscus	gi|383075026|gb|AJFE01003225.1|	2	261	1655	2419	7.21E-111	194
capsid triplex subunit 2	Pan paniscus	gi|383075026|gb|AJFE01003225.1|	1	304	23468	24373	4.02E-102	210
DNA packaging terminase subutnit 1	Pan paniscus	gi|383075026|gb|AJFE01003225.1|	447	822	20374	21441	1.97E-67	158
DNA packaging terminase subutnit 1	Pan paniscus	gi|383075026|gb|AJFE01003225.1|	304	447	16619	17050	6.26E-14	50
DNA packaging prot UL32	Pan paniscus	gi|383069699|gb|AJFE01006050.1|	228	346	3387	3752	3.84E-06	35
DNA packaging prot UL32	Pan paniscus	gi|383069699|gb|AJFE01006050.1|	620	716	2440	2721	0.162857	30
DNA packaging terminase subutnit 1	Pan paniscus	gi|383075026|gb|AJFE01003225.1|	301	668	20377	21480	6.76E-88	169
DNA packaging terminase subutnit 1	Pan paniscus	gi|383075026|gb|AJFE01003225.1|	48	300	16256	17050	1.45E-23	77
ATPase subunit terminase	Pan paniscus	gi|383075026|gb|AJFE01003225.1|	331	467	20383	20784	9.05E-05	43
hypothetical protein	Pan paniscus	gi|383075026|gb|AJFE01003225.1|	40	893	28569	30845	2.60E-157	372
uracil DNA glycosylase	Pan paniscus	gi|383075026|gb|AJFE01003225.1|	2	261	1655	2419	7.21E-111	194
capsid triplex subunit 2	Pan paniscus	gi|383075026|gb|AJFE01003225.1|	1	304	23468	24373	4.02E-102	210
DNA packaging terminase subutnit 1	Pan paniscus	gi|383075026|gb|AJFE01003225.1|	447	822	20374	21441	1.97E-67	158
DNA packaging terminase subutnit 1	Pan paniscus	gi|383075026|gb|AJFE01003225.1|	304	447	16619	17050	6.26E-14	50
DNA packaging prot UL32	Pan paniscus	gi|383069699|gb|AJFE01006050.1|	228	346	3387	3752	3.84E-06	35
DNA packaging prot UL32	Pan paniscus	gi|383069699|gb|AJFE01006050.1|	620	716	2440	2721	0.162857	30
DNA packaging terminase subutnit 1	Pan paniscus	gi|383075026|gb|AJFE01003225.1|	301	668	20377	21480	6.76E-88	169
DNA packaging terminase subutnit 1	Pan paniscus	gi|383075026|gb|AJFE01003225.1|	48	300	16256	17050	1.45E-23	77
DNA packaging terminase subutnit 1	Tarsius syrichta	gi|202855770|gb|ABRT010424292.1|	315	405	4733	5002	1.74E-07	34
tegument protein UL16	Tarsius syrichta	gi|202855770|gb|ABRT010424292.1|	107	270	5379	5843	4.48E-18	60
tegument protein UL16	Tarsius syrichta	gi|202855770|gb|ABRT010424292.1|	308	356	5138	5281	4.48E-18	22
tegument protein UL16	Tarsius syrichta	gi|202593353|gb|ABRT010686709.1|	11	131	3	347	3.75E-06	52
DNA packaging terminase subutnit 1	Tarsius syrichta	gi|202855770|gb|ABRT010424292.1|	171	259	4736	5002	5.02E-67	62
DNA packaging terminase subutnit 1	Tarsius syrichta	gi|202855770|gb|ABRT010424292.1|	1	172	4230	4742	5.02E-67	73
DNA packaging terminase subutnit 1	Tarsius syrichta	gi|202855770|gb|ABRT010424292.1|	257	300	4993	5121	5.02E-67	27
DNA packaging terminase subutnit 1	Tarsius syrichta	gi|202855770|gb|ABRT010424292.1|	315	405	4733	5002	1.74E-07	34
tegument protein UL16	Tarsius syrichta	gi|202855770|gb|ABRT010424292.1|	107	270	5379	5843	4.48E-18	60
tegument protein UL16	Tarsius syrichta	gi|202855770|gb|ABRT010424292.1|	308	356	5138	5281	4.48E-18	22
tegument protein UL16	Tarsius syrichta	gi|202593353|gb|ABRT010686709.1|	11	131	3	347	3.75E-06	52
DNA packaging terminase subutnit 1	Tarsius syrichta	gi|202855770|gb|ABRT010424292.1|	171	259	4736	5002	5.02E-67	62
DNA packaging terminase subutnit 1	Tarsius syrichta	gi|202855770|gb|ABRT010424292.1|	1	172	4230	4742	5.02E-67	73
DNA packaging terminase subutnit 1	Tarsius syrichta	gi|202855770|gb|ABRT010424292.1|	257	300	4993	5121	5.02E-67	27
