# Supplementary material for: The First Endogenous Herpesvirus, Identified in the Tarsier Genome, and Novel Sequences from Primate Rhadinoviruses and Lymphocryptoviruses
Source: PLoS Genet. 2014 Jun 19;10(6):e1004332. doi: 10.1371/journal.pgen.1004332 (PMC4063692; doi:10.1371/journal.pgen.1004332)
Supplement: Table S3 — The spreadsheet contains a catalogue of reciprocal BLASTx results. Shortlisted viral contigs were checked against protein databases in order to eliminate false positive results from earlier rounds of BLAST searching. Information for each of the viruses investigated in this study is found on separate sheets. Sheet 1: DmadHVLs, sheet 2: PpanHVLs, sheet 3: TsyrHVLs. ‘Top Alternative Hit’ documents the best non-viral BLAST hit if the best hit is viral, or else shows the top viral hit if the best hit is host. The latter scenario occurs for a false positive hit. (PDF) [file pgen.1004332.s005.pdf]

Primary Result							Top alternative hit				
**Query**	**length (bp)**	**Species**	**Description**	**Hit ID**	**E-value**	**Coverage**	**Species**	**Description**	**Hit ID**	**E-value**	**Coverage**
AGTM010394531.1	2006	Saimiriine herpesvirus 2	major capsid protein	NP_040227.1	0	96%	Vibrio anguillarum	Topoisomerase IV subunit A	YP_004565394.1	0.48	40%
			hypothetical protein	NP_040226.1	1.34E-28	96%	None				
AGTM010049177.1	475	Equid herpesvirus 2	ORF 29a	NP_042630.1	7.00E-23	82%	Thermus sp.	Methyltransferase	YP_001966068.1	4.2	31%
AGTM010086645.1	329	Bovine herpesvirus 4	hypothetical protein	NP_076558.1	4.00E-36	99%	Branchiostoma floridae	hypothetical protein	XP_002601625.1	36.6	28%
AGTM010130173.1	613	Saimiriine herpesvirus 2	hypothetical protein	NP_040226.1	1.00E-37	95%	Saprospira grandis	hypothetical protein	YP_005320966.1	4.3	30%
AGTM010365164.1	4430	Ovis aries	thymidylate synthase	XP_004020543.1	2.00E-26	5%	European catfish virus	thymidylate synthase	YP_006347622.1	3.00E-20	5%
AGTM010394582.1	1925	Mus musculus	thymidylate synthase	NP_067263.1	2.00E-175	46%	European catfish virus	thymidylate synthase	YP_006347622.1	2.00E-161	45%
AGTM010394559.1	1236	Equid herpesvirus 2	ORF 68	NP_042667.1	3.00E-85	90%	Methylibium petroleiphilum	ferredoxin	YP_001020565.1	8.7	9%
			ORF 69	NP_042668.1	3.00E-14						
AGTM010394565.1	1059	Bovine herpesvirus 4	major capsid protein	NP_076517.1	6.00E-120	96%	Bacteroides stercoris	hypothetical protein	ZP_02435406.1		
		Saimiriine herpesvirus 2	hypothetical protein	NP_040228.1	1.00E-48						
AGTM010394574.1	1107	Equid herpesvirus 2	Helicase-primase complex	NP_042653.1	1.00E-127	99%	None				
AGTM010394781.1	1125	Equid herpesvirus 2	ribonucleotide reductase	NP_042657.1	1.65E-154	81%	Naegleria gruberi	predicted protein	XP_002680453.1	1.00E-32	72%
AGTM010394600.1	1794	Human herpesvirus 8	latency antigen	YP_001129431.1	4.00E-13	22%	None				
AGTM010394632.1	2640	Bovine herpesvirus 4	DNA replication protein	NP_076551.1	1.00E-57	76%	Ornithorhynchus anatinus	fibrocystin-like	XP_003428622.1	4.6	12%
		Ateline herpesvirus 3	ORF 58	NP_048029.1	5.00E-47						
AGTM010394693.1	3229	Human herpesvirus 8	ORF 49	YP_001129402.1	2.00E-62	53%	Burkholderia multivorans	PF06906 family protein	ZP_15925465.1	1.3	3%
		Saimiriine herpesvirus 2	R transactivator	NP_040252.1	2.00E-55		Bos taurus	zinc finger protein	XP_003587411.1	3.7	6%
AGTM010394708.1	1234	Ateline herpesvirus 3	ORF 29a	NP_048006.1	7.00E-100	102% (overlap)	Synechococcus phage	terminase	YP_007001615.1	2.3	23%
		Bovine herpesvirus 4	hypothetical protein	NP_076525.1	7.00E-75						
AGTM010394888.1	1775	Ateline herpesvirus 3	ORF 48	NP_048021.1	5.00E-29	48%	None				
AGTM010394834.1	1133	Equid herpesvirus 2	glycoprotein H	NP_042618.1	1.00E-27	71%	Trichomonas vaginalis	polymerase zeta subunit	XP_001326973.1	4.3	20%
		Saimiriine herpesvirus 2	DNA polymerase	NP_040211.1	8.00E-21						
AGTM010394847.1	701	Macacine herpesvirus 5	tegument protein	NP_570811.1	1.00E-60	98%	Nematostella vectensis	predicted protein	XP_001634054.1	0.78	28%
AGTM010394854.1	4653	Saimiriine herpesvirus 2	unnamed protein	NP_040222.1	2.00E-53	90%	Dickeya dadantii	ribosome-associated factor	YP_003881078.1	4.5	3%
			unnamed protein	NP_040221.1	6.00E-163						
			unnamed protein	NP_040220.1	5.00E-80						
			unnamed protein	NP_040219.1	5.00E-79						
AGTM010394873.1	2266	Bovine herpesvirus 4	ssDNA-binding protein	NP_040208.1	0	98%	Metaseiulus occidentalis	NCOAT-like protien	XP_003743615.1	2.1	26%
		Saimiriine herpesvirus 2	transport protein	NP_040209.1	4.29E-20						
AGTM010394913.1	782	Bovine herpesvirus 4	FGAM-synthetase	NP_076568.1	1.00E-13	49%	Callithrix jacchus	dehydrogenase	XP_002760484.2	9.9	62%
AGTM010395291.1	1725	Ateline herpesvirus 3	tegument protein	NP_048035.1	5.00E-13	35%	Megavirus lba	collagen-like protein 2	YP_007418171.1	3.3	15%
AGTM010394923.1	2334	Saimiriine herpesvirus 2	alkaline exonuclease	NP_040239.1	6.33E-119	98%	Tribolium castaneum	CG11489-like	XP_968798.1	0.018	27%
		Equid herpesvirus 2	virion protein kinase	NP_042633.1	7.00E-87						
AGTM010395004.1	1221	Saimiriine herpesvirus 2	hypothetical protein	NP_040212.1	1.00E-39	92%	Rhizobium sp	nidazolesuccinocarboxamide (SA	ZP_10732526.1	2.8	19%
			hypothetical protein	NP_040213.1	2.00E-14						
AGTM010395025.1	1038	Equid herpesvirus 2	ORF 55	NP_042652.1	1.00E-76	103% (overlap)	None				
			Helicase-primase complex	NP_042653.1	6.00E-26						
AGTM010395096.1	2107	Bovine herpesvirus 4	hypothetical protein	NP_076544.1	4.01E-25	19%	None				
		Equid herpesvirus 2	ORF 53	NP_042650.1	0.89						
AGTM010401350.1	1336	Bovine herpesvirus 4	hypothetical protein	NP_076516.1	1.11E-55	96%	Naumovozyma castellii	hypothetical protein	XP_003675768.1	2.1	13%
		Macacine herpesvirus 5	hypothetical protein	NP_570764.1	1.00E-53						
AGTM010395359.1	533	Equid herpesvirus 2	glycoprotein M	NP_042636.1	1.00E-62	87%	None				
AGTM010395382.1	867	Bovine herpesvirus 4	tegument protein	NP_076556.1	5.00E-37	98%	Bifidobacterium animalis	hypothetical protein	YP_006279945.1	1.4	22%
AGTM010395445.1	1504	Saimiriine herpesvirus 2	ribonucleotide reductase	NP_040263.1	2.00E-127	95%	None				
			hypothetical protein	NP_040264.1	3.00E-31						
AGTM010395446.1	1115	Bovine herpesvirus 4	se-primase complex comp	NP_076532.1	3.66E-46	81%	None				
AGTM010456527.1	722	Equid herpesvirus 2	capsid protein	NP_042621.1	2.00E-107	71%	None				
AGTM010446062.1	394	Ateline herpesvirus 3	large tegument protein	NP_048035.1	3.00E-16	95%	Bacteroides fragilis	hypothetical protein	ZP_17249539.1	8.1	57%
AGTM010447026.1	1203	Equid herpesvirus 2	glycoprotein M	NP_042636.1	1.00E-67	88%	None				
			deoxyribonuclease	NP_042634.1	5.65E-18						
		Saimiriine herpesvirus 2	unnamed protein	NP_040240.1	1.00E-03						
AGTM010448489.1	710	Equid herpesvirus 2	tegument protein	NP_042661.1	2.00E-45	90%					
AGTM010448782.1	714	Macacine herpesvirus 5	tegument protein	NP_570811.1	1.06E-46	98%	Lactobacillus gigeriorum	1-phosphofructokinase	ZP_10417669.1	2.2	27%
AGTM010448810.1	323	felis catus	transferase	XP_003985863.1	8.00E-60	99%	None				
AGTM010455736.1	717	Saimiriine herpesvirus 2	hypothetical protein	NP_040266.1	3.00E-44	97%	Chlorobium limicola	maf protein	YP_001943015.1	2.1	37%
AGTM010461791.1	440	Ateline herpesvirus 3	ORF 34	NP_048007.1	9.00E-39	98%	Brevibacillus laterosporus	SNF2 family domain protein	ZP_16303839.1	2.9	36%
AGTM010479572.1	815	Ateline herpesvirus 3	DNA polymerase	NP_047983	6.42E-132	98%	Anolis carolinensis	DNA polymerase subunit-like	XP_003222764.1	2.00E-52	96%
AGTM010462116.1	644	Ateline herpesvirus 3	ORF 34	NP_048007.1	3.00E-18	90%	Glaciecola pallidula	hypothetical protein	ZP_11333206.1	0.053	41%
			ORF 35	NP_048008.1	3.00E-19						
AGTM010463876.1	1011	Saimiriine herpesvirus 2	ssDNA-binding protein	NP_040208.1	4.00E-117	100%	Desulfovibrio hydrothermalis	DNA gyrase	YP_007325172.1	0.057	21%
		Bovine herpesvirus 4	transport protein	NP_076499.1	1.12E-53	98%	None				
AGTM010473543.1	503	Bovine herpesvirus 4	v-FGAM-synthase	NP_076495.1	6.00E-12	96%	Actinobacillus pleuropneumoniae	hypothetical protein	ZP_07538257.1	1.7	88%
AGTM010635235.1	618	Callitrichine herpesvirus 3	ORF23	NP_733876.1	1.00E-20	99%	Branchiostoma floridae	hypothetical protein	XP_002590671.1	3.2	24%
		Human herpesvirus 8	ORF32	YP_001129385.1	4.00E-14						
AGTM010480454.1	515	Ovine herpesvirus 2	ORF 22	YP_438146.1	1.70E-01	97%					
			ORF 23	YP_438147.1	5.00E-28		Leptospira biflexa	permease	YP_001838069.1	0.36	43%
AGTM010486910.1	379	Human herpesvirus 8	ORF61	YP_001129418.1	3.00E-37	96%	Enterocytozoon bieneusi	onucleoside-diphosphate reduct	XP_002650066.1	3.00E-10	91%
AGTM010493322.1	678	Equid herpesvirus 2	Helicase-primase	NP_042653.1	9.00E-67	84%	Azospirillum brasilense	phosphohydrolase	YP_005032122.1	1.1	20%
AGTM010496494.1	500	Saimiriine herpesvirus 3	envelope glycoprotein B	YP_004940228.1	2.00E-29	95%	Cucumis sativus	nucleosidase 1-like	XP_004144832.1	2.7	57%
AGTM010515626.1	596	Equid herpesvirus 2	ORF 54	NP_042651.1	2.00E-46	79%	Hydrogenobaculum sp.	triphosphate deaminase	YP_007499923.1	0.015	32%
AGTM010547017.1	622	Bovine herpesvirus 4	hypothetical protein	NP_076516.1	8.00E-96	98%	Neisseria elongata glycolytica	hypothetical protein	ZP_06735599.1	3.00E-23	28%
AGTM010633131.1	315	Saimiri boliviensis boliviensis	interleukin-17A	XP_003923160.1	8.00E-43	74%	None				
AGTM010731808.1	672	Bovine herpesvirus 4	randed DNA-binding protei	NP_076498.1	1.00E-18	65%	Alteromonas macleodii	SecY interacting protein	YP_006797974.1	0.52	24%
		Equid herpesvirus 2	ORF E4	NP_042601.1	1.00E-07						
AGTM010653738.1	269	Ateline herpesvirus 3	ORF 63	NP_048034.1	3.00E-19	90%	None				
AGTM010659043.1	946	Equid herpesvirus 2	tegument protein	NP_042672.1	5.70E-24	23%	Desulfotalea psychrophila	horibosylformylglycinamidine sy	YP_064440.1	3.00E-14	21%
AGTM010668737.1	422	Ateline herpesvirus 3	major capsid protein	NP_047996.1	5.00E-51	96%	Haloarcula japonica	conjugation protein	ZP_21693618.1	0.39	69%
AGTM010722312.1	672	Ateline herpesvirus 3	ORF 62	NP_048033.1	4.16E-70	86%	Providencia rettgeri	lycoside hydrolase family protei	ZP_16353551.1	0.53	40%
AGTM010722683.1	464	Bovine herpesvirus 4	ribonucleoide reductase	NP_076553.1	2.68E-59	99%	Encephalitozoon intestinalis	onucleoside diphosphate reduct	XP_003073772.1	2.00E-16	73%
AGTM010723649.1	312	Saimiriine herpesvirus 2	glycoprotein H	NP_040224.1	6.00E-33	99%	Meleagris gallopavo	contactin-3-like	XP_003210246.1	1.8	81%
AGTM011564346.1	10759	Homo sapiens	thymidylate synthase	NP_001062.1	3.00E-38	7%	European catfish virus	thymidylate synthase	YP_006347622.1	8.00E-24	5%
AGTM010808082.1	592	Saimiriine herpesvirus 2	unnamed protein	NP_040265.1	4.60E-13	69%	Physcomitrella patens	predicted protein	XP_001763433.1	0.21	46%
AGTM010904390.1	243	Macacine herpesvirus 5	DNA replication protein	NP_570796.1	1.12E-20	98%	Photobacterium profundu	bacteriophage integrase	YP_133335.1	5.7	48%
AGTM011204162.1	260	Saimiriine herpesvirus 2	major capsid protein	NP_040227.1	7.00E-40	96%	Paenibacillus dendritiformis	group 1 glycosyl transferase	ZP_09677647.1	1.4	31%
AGTM011244974.1	212	Otolemur garnettii	-glycoprotein beta-1,6-N-ac	XP_003787766.1	8.00E-38	99%	None				
AGTM011531363.1	4716	Otolemur garnettii	-glycoprotein beta-1,6-N-ac	XP_003784439.1	0	27%	None				
AGTM011536785.1	19290	Macaca mulatta	nembrane glycoprotein pre	NP_001244473.1	5.00E-56	3%	Lumpy skin disease virus	DV138 Ig domain OX-2-like prote	NP_150572.1	4.00E-04	1%
AGTM011537755.1	8267	Equus caballus	nidylate synthase-like isofo	XP_001492275.1	3.07E-29	6%	European catfish virus	thymidylate synthase	YP_006347622.1	2.00E-24	6%
AGTM011544182.1	8417	Nomascus leucogenys	type A, EGF and pentraxin	XP_003260519.1	0	90%	None				
AGTM011552975.1	8104	Otolemur garnettii	omplement receptor type	XP_003792361.1	9.00E-68	24%	Cowpox virus	CPXV034	NP_619823.1	1.00E-13	12%
AGTM011563296.1	6290	Otolemur garnettii	interleukin-17A	XP_003789688.1	1.00E-40	6%	None				
AGTM011636650.1	4630	Otolemur garnettii	glycoprotein	XP_003781590.1	2.00E-25	5%	None				
AGTM011564795.1	5624	Pan troglodytes	oprotein beta-1,6-N-acetyl	XP_001145936.1	1.00E-131	11%	None				
AGTM011579171.1	12618	Sus scrofa	membrane glycoprotein	XP_003483369.1	2.99E-58	5%	Goatpox virus Pellor	hypothetical protein	YP_001293329.1	0.002	2%
AGTM011602539.1	18693	Callithrix jacchus	-glycoprotein beta-1,6-N-ac	XP_002747748.1	0	6%	None				
AGTM011606314.1	17433	Callithrix jacchus	l-glycoprotein beta-1,6-N-a	XP_002742885.1	2.00E-128	6%	Bovine herpesvirus 4	ta-1,6-N-acetylglucosaminyltran	NP_076572.1	1.00E-80	5%
AGTM011611918.1	7152	Otolemur garnettii	thymidylate synthase	XP_003784845.1	5.00E-128	11%	Cercopithecine herpesvirus 9	thymidylate synthase	NP_077428.1	2.00E-94	11%
AGTM011618091.1	2656	Papio anubis	interleukin-17F	XP_003897778.1	2.07E-33	8%	Saimiriine herpesvirus 2	unnamed protein product	NP_040215.1	3.00E-20	8%
AGTM011629352.1	5409	Ailuropoda melanoleuca	-1,6-N-acetylglucosaminyl-	XP_002915964.1	2.00E-99	10%	Bovine herpesvirus 4	ta-1,6-N-acetylglucosaminyltran	NP_076572.1	3.00E-40	10%
AGTM011769130.1	1954	Ovis aries	membrane cofactor	XP_004013973.1	2.00E-36	29%	Saimiriine herpesvirus 2	dihydrofolate reductase	NP_040203.1	6.00E-08	1%
AGTM011643537.1	5770	Loxodonta africanus	dihydrofolate reductase	XP_003408165.1	6.00E-11	1%	Saimiriine herpesvirus 2	dihydrofolate reductase	NP_040203.1	6.00E-08	1%
AGTM011679335.1	3436	Nomascus leucogenys	oprotein beta-1,6-N-acetyl	XP_003267479.1	1.00E-56	11%	Bovine herpesvirus 4	ta-1,6-N-acetylglucosaminyltran	NP_076572.1	8.00E-12	7%
AGTM011703833.1	3762	Saimiriine herpesvirus 2	helicase	NP_040246.1	0	67%	Hydra magnipapillata	uracil-DNA glycosylase-like	XP_002166406.1	2.00E-59	18%
		Ateline herpesvirus 3	hypothetical protein	NP_048019.1	3.00E-108						
AGTM011704110.1	1165	Bovine herpesvirus 4	v-FGAM-synthase	NP_076495.1	3.00E-60	95%	atus Kinetoplastibacterium desouzaii T	horibosylformylglycinamidine sy	YP_007459298.1	3.00E-16	69%
AGTM011717059.1	1750	Human herpesvirus 8	Glycoprotien B	YP_001129354.1	8.00E-104	89%	Escherichia coli KTE163	hypothetical protein WKG_03335		7.8	9%
		Saimiriine herpesvirus 2	transport protein	NP_040209.1	7.80E-83						
AGTM011718482.1	5084	felis catus	transposable element der	XP_003990972.1	3.00E-40	10%	Saimiriine herpesvirus 2	dihydrofolate reductase	NP_040203.1	2.00E-09	2%
		European catfish virus	dihydrofolate reductase	YP_006347707.1	2.00E-12						
AGTM011750305.1	2680	Otolemur garnettii	uncharacterized protein	XP_003788410.1	0	34%	Bovine herpesvirus 4	ta-1,6-N-acetylglucosaminyltran	NP_076572.1	4.00E-71	26%
AGTM011754680.1	5122	Otolemur garnettii	-glycoprotein beta-1,6-N-ac	XP_003785965.1	0	26%	lephant endotheliotropic herpesvirus 1	ta-Acetyl-Glucosamine Transfera	YP_007517197.1	1.00E-99	22%
AGTM011876226.1	3319	Otolemur garnettii	chemokine receptor	XP_003785015.1	0	32%	None	None			
AGTM011771171.1	7616	Otolemur garnettii	omplement receptor type	XP_003792361.1	2.00E-64	26%	Macacine herpesvirus 5	complement binding protein	NP_570746.1	2.00E-09	10%
AGTM011771918.1	968	Bovine herpesvirus 4	FGAM-synthetase	NP_076568.1	9.00E-78	99%	Drosophila grimshawi	GH10940	XP_001988021.1	8.00E-16	87%
AGTM011792607.1	4739	Otolemur garnettii	dihydrofolate reductase	XP_003786000.1	7.00E-31	4%	Saimiriine herpesvirus 2	dihydrofolate reductase	NP_040203.1	1.00E-22	4%
AGTM011819513.1	1408	Bovine herpesvirus 4	hypothetical protein	NP_076568.1	7.00E-77	36%	Anolis carolinensis	ribosylformylglycinamidine synt	XP_003227285.1	3.00E-08	72%
AGTM011819567.1	1009	Saimiriine herpesvirus 2	unnamed protein	NP_040244.1	5.00E-61	100%	Nasonia vitripennis	-dependent transcription factor ATF-2-like			
		Macaca fuscata rhadinovirus	Helicase-primase complex	NP_042638.1	1.00E-06				XP_001603069.1	0.88	29%
		Saimiri boliviensis boliviensis	-glycoprotein beta-1,6-N-ac	XP_003927479.1	5.00E-157		Bovine herpesvirus 4	ta-1,6-N-acetylglucosaminyltran	NP_076572.1	7.00E-55	31%
AGTM011835354.1	3893	Otolemur garnettii	type A, EGF and pentraxin	XP_003785430.1	4.00E-132	25%	None	None			
AGTM011914563.1	5415	Ailuropoda melanoleuca	thetical protein PANDA_00	XP_002915951.1	1.00E-176	17%	Bovine herpesvirus 4	ta-1,6-N-acetylglucosaminyltran	NP_076572.1	2.00E-65	13%
AGTM012005779.1	284	Saimiri boliviensis boliviensis	-glycoprotein beta-1,6-N-ac	XP_003927479.1	3.25E-38	99%	Bovine herpesvirus 4	ta-1,6-N-acetylglucosaminyltran	NP_076572.1	1.00E-12	58%
AGTM011921304.1	364	Oryctolagus cuniculus	thymidylate synthase	XP_002713484.1	1.17E-57	80%	Cercopithecine herpesvirus 9	thymidylate synthase	NP_077428.1	5.00E-47	80%
AGTM011948974.1	2508	Ateline herpesvirus 3	pothetical protein AtHV3gp	NP_048000.1	0	89%					
			pothetical protein AtHV3gp	NP_048003.1	1.01E-60		Branchiostoma floridae	thetical protein BRAFLDRAFT_10	XP_002591195.1	0.081	14%
		Bovine herpesvirus 4	unnamed protein	NP_076522.1	1.00E-07		Physcomitrella patens subsp. patens	predicted protein	XP_001766999.1	1.8	5%
			DNA packaging	NP_076524.1	1.24E-05						
AGTM011952769.1	683	Equus caballus	brand factor type A, EGF ar	XP_001916184.1	2.37E-142	99%	Macacine herpesvirus 5	complement binding protein	NP_570746.1	2.00E-14	99%
AGTM011994144.1	440	Nomascus leucogenys	type A, EGF and pentraxin	XP_003260519.1	1.00E-84	99%	Deerpox virus W-848-83	EEV host range protein	YP_227531.1	1.00E-12	90%
AGTM012314371.1	663	Equid herpesvirus 2	ORF 54	NP_042651.1	1.79E-49	64%	acterium catenulatum DSM 16992 = JC	ypothetical protein BIFCAT_0055	ZP_03323783.1	2.00E-04	62%
AGTM012015256.1	301	Ateline herpesvirus 3	transport protein	NP_047981.1	4.30E-25	99%	Pan troglodytes	reonine synthase-like 1 isoform	XP_521671.1	1.8	36%
AGTM012090034.1	217	Ateline herpesvirus 3	mitogen	NP_047986.1	3.02E-12	66%	Synechococcus sp. WH 8016	dehalogenase domain protein h	ZP_08957352.1	9.3	73%
AGTM012095796.1	210	Ateline herpesvirus 3	mitogen	NP_047986.1	1.52E-14	78%	Ralstonia solanacearum K60-1	ialdehyde dehydrogenase I,NAD	ZP_16319481.1	2.5	48%
AGTM012163629.1	216	Ateline herpesvirus 3	mitogen	NP_047986.1	5.72E-11	66%	Microcystis aeruginosa PCC 9808	Modification methylase HindV	ZP_18841800.1	2.2	97%
AGTM012291937.1	3322	Ailuropoda melanoleuca	-1,6-N-acetylglucosaminyl-	XP_002915964.1	4.61E-175	28%	Bovine herpesvirus 4	ta-1,6-N-acetylglucosaminyltran	NP_076572.1	3.00E-67	21%
AGTM012314139.1	1906	Saimiriine herpesvirus 2	unnamed protein	NP_040204.1	2.95E-99	67%	Trichinella spiralis	horibosylformylglycinamidine sy	XP_003372964.1	1.00E-21	64%
AGTM012580127.1	234	Mus musculus	thymidylate synthase	NP_067263.1	6.00E-24	60%	Human herpesvirus 8	ORF70	YP_001129361.1	9.00E-17	58%
AGTM012318916.1	602	Human herpesvirus 8	ORF 22	YP_001129375.1	2.24E-27	91%	Cellulophaga algicola DSM 14237	hypothetical protein	YP_004163066.1	3.1	17%
AGTM012354265.1	606	Ovine herpesvirus 2	ORF 11	YP_438139.1	3.00E-26	100%	None	None			
AGTM012375206.1	744	Saimiriine herpesvirus 2	unnamed protein	NP_040229.1	3.00E-15	58%	Pseudomonas putida DOT-T1E	409 family phage-associated pro	YP_006532001.1	0.35	16%
AGTM012418162.1	341	Ateline herpesvirus 3	mitogen	NP_047986.1	5.43E-10	48%	Mouse mammary tumor virus	Pr48	NP_056884.1	0.41	24%
AGTM012969727.1	800	Ateline herpesvirus 3	hypothetical protein	NP_047997.1	5.70E-80	84%	Tetrahymena thermophila	othetical protein TTHERM_00066	XP_001007710.1	0.61	45%
		Human herpesvirus 8	ORF 27	YP_001129380.1	8.00E-82		Leadbetterella byssophila DSM 17132	hypothetical protein Lbys_1126	YP_003997201.1	2.1	18%
AGTM012703695.1	215	Saimiri boliviensis boliviensis	-1,6-N-acetylglucosaminyl-	XP_003927404.1	4.26E-36	99%	Bovine herpesvirus 4	ta-1,6-N-acetylglucosaminyltran	NP_076572.1	1.00E-19	94%
AGTM012815981.1	202	Macaca mulatta	dylate synthetase, isoform	NP_001182436.1	2.00E-29	92%	Human herpesvirus 3	thymidylate synthase	NP_040136.1	2.00E-13	65%
AGTM012840725.1	252	Papio anubis	dihydrofolate reductase-like	XP_003893111.1	1.27E-47	98%	Saimiriine herpesvirus 2	dihydrofolate reductase	NP_040203.1	1.00E-38	97%
AGTM012927422.1	2254	Anolis carolinensis	thymidylate synthase-like	XP_003219704.1	1.69E-18	51%	Human herpesvirus 3	thymidylate synthase	NP_040136.1	3.00E-16	19%
		Plasmodium yoelii yoelii	hypothetical protein	XP_726997.1	2.00E-11						
AGTM012945613.1	1348	Bovine herpesvirus 4	unnamed protein	NP_076560.1	5.00E-13	83%	None				
			tegument protein	NP_076559.1	4.00E-75						
			probable glycoprotein	NP_076561.1	4.00E-42						
AGTM012948373.1	818	Saimiriine herpesvirus 2	unnamed protein	NP_040268.1	9.00E-06	66%	None				
AGTM012954910.1	593	Saimiri boliviensis boliviensis	N-acetylglucosaminyl-trans	XP_003927402.1	1.68E-68	66%	Bovine herpesvirus 4	ta-1,6-N-acetylglucosaminyltran	NP_076572.1	5.00E-14	30%
AGTM013180837.1	312	Macaca mulatta	dihydrofolate reductase	XP_001110551.2	8.90E-42	74%	Macacine herpesvirus 5	dihydrofolate reductase	NP_570745.1	6.00E-16	49%
AGTM012985849.1	510	Macacine herpesvirus 5	large tegument protein	NP_570812.1	5.00E-21	88%	Bacteroidetes oral taxon 274 str. F0058	eptidyl-prolyl cis-trans isomerase	ZP_06982726.1	0.19	35%
AGTM012997089.1	1272	Saimiriine herpesvirus 2	IE-G	NP_040216.1	5.84E-24		None				
		Gorilla Gorilla	tigger-TE	XP_004033412.1	8.00E-14						
AGTM013034944.1	517	Macacine herpesvirus 5	hypothetical protein	NP_570817.1	6.78E-59	99%	Cavia porcellus	myosin-XV-like	XP_003466009.1	1.8	23%
AGTM013041174.1	475	Bovine herpesvirus 4	DNA polymerase	NP_076501.1	1.75E-63	99%	thanomethylovorans hollandica DSM 15	DNA polymerase	YP_007301837.1	8.00E-10	96%
AGTM013121095.1	244	Bovine herpesvirus 4	hypothetical protein	NP_076558.1	8.57E-13	71%	None				
AGTM013080733.1	277	Papio anubis	,6-N-acetylglucosaminyl-tra	XP_003897091.1	2.00E-57	99%	Bovine herpesvirus 4	ta-1,6-N-acetylglucosaminyltran	NP_076572.1	7.00E-31	97%
AGTM013133622.1	220	Pan troglodytes	eta-1,6-N-acetylglucosami	XP_003311128.1	2.72E-39	98%	Bovine herpesvirus 4	ta-1,6-N-acetylglucosaminyltran	NP_076572.1	3.00E-24	98%

Primary Hit							Top Alternative Hit				
**Query ID**	**Length**	**Species**	**Description**	**Hit ID**	**E-value**	**coverage**	**Species**	**Description**	**Hit ID**	**E-value**	**coverage**
AJFE01001036.1	4833	Pan paniscus	viral interleukin-10	XP_003804206.1	4.00E-94	10	Human Herpesvirus 4	BCRF1	YP_001129439.1	9.00E-78	10
AJFE01001973.1	1331	Human herpesvirus 4 type 2	BOLF1	YP_001129450.1	4.00E-178	78	Halorubrum tebenquichense	Bacterio-opsin activator	ZP_21586426.1	1.8	14
AJFE01002280.1	1014	Human herpesvirus 4 type 2	BOLF1	YP_001129450.1	1.19E-102	75	NONE				
AJFE01001185.1	7204	Human herpesvirus 4	BALF2	YP_401717.1	0.00E+00	80	Pseudovibrio sp.	utative membrane associated protei	YP_005079425.1	3.2	41
		Human herpesvirus 4	BARF1	YP_401719.1	4.00E-107						
		Human herpesvirus 4	BALF1	YP_401718.1	2.00E-104						
		Human herpesvirus 4	BALF3	YP_401715.1	1.00E-95						
		Human herpesvirus 4	BNLF2b	YP_401720.1	1.00E-21						
AJFE01005652.1	4766	Macacine herpesvirus 4	BALF4	YP_068009.1	0.00E+00	98	Acyrthosiphon pisum	DNA polymerase	XP_001948892.2	4.00E-10	10
		Human herpesvirus 4 type 2	BALF5	YP_001129507.1	0.00E+00						
		Macacine herpesvirus 4	BALF3	YP_001129508.1	0.00E+00						
AJFE01003225.1	44035	Human herpesvirus 4	BKRF3	YP_401679.1	2.00E-160	100	None				
		Human herpesvirus 4	BBLF4	YP_401681.1	0.00E+00						
		Human herpesvirus 4 type 2	BBRF1	YP_001129476.1	0.00E+00						
		Human herpesvirus 4	BBRF2	YP_401683.1	5.00E-169						
		Human herpesvirus 4	BBLF2/BBLF3	YP_401684.1	0.00E+00						
		Human herpesvirus 4 type 2	BBRF3	YP_001129479.1	0.00E+00						
		Human herpesvirus 4 type 2	BGLF5	YP_001129481.1	0.00E+00						
		Human herpesvirus 4	BGLF4	YP_401688.1	0.00E+00						
		Human herpesvirus 4	BGLF3	YP_401689.1	0.00E+00						
		Human herpesvirus 4	BGRF1/BDRF1	YP_401690.1	2.90E-179						
		Human herpesvirus 4	BGLF2	YP_401691.1	0.00E+00						
		Human herpesvirus 4 type 2	BGLF1	YP_001129487.1	0.00E+00						
		Human herpesvirus 4	BDLF4	YP_401693.1	2.00E-133						
		Human herpesvirus 4	BGRF1/BDRF1	YP_401690.1	0.00E+00						
		Human herpesvirus 4	BDLF2	YP_401695.1	2.00E-173						
		Macacine herpesvirus 4	BDLF1	YP_067993.1	5.30E-179						
		Human herpesvirus 4	BcLF4	YP_401697.1	0.00E+00						
		Human herpesvirus 4	BcRF1	YP_401698.1	0.00E+00						
		Human herpesvirus 4	BTRF1	YP_401699.1	0.00E+00						
		Human herpesvirus 4	BXLF2	YP_401700.1	0.00E+00						
		Human herpesvirus 4	BXLF1	YP_401701.1	0.00E+00						
		Human herpesvirus 4 type 2	BXRF1	YP_001129498.1	3.00E-144						
		Human herpesvirus 4	BVRF1	YP_401703.1	0.00E+00						
		Human herpesvirus 4 type 2	BVLF1	YP_001129500.1	4.00E-136						
		Human herpesvirus 4	BVRF2	YP_401704.1	0.00E+00						
		Human herpesvirus 4	membrane glycoprotein	YP_401706.1	8.30E-98						
AJFE01004538.1	12095	Human herpesvirus 4	BPLF1	YP_401652.1	0.00E+00	71	None				
		Human herpesvirus 4 type 2	BFRF2	YP_001129447.1	0.00E+00	14					
		Human herpesvirus 4	BFRF1	YP_401649.1	1.00E-91	5					
AJFE01121077.1	1720	Human herpesvirus 4	BHRF1	YP_401646.1	8.00E-94	33	Microlunatus phosphovorus	hypothetical protein	YP_004571555.1	0.62	22
AJFE01006050.1	5011	Human herpesvirus 4	BHRF1	YP_401646.1	6.10E-55	7	None				
		Human herpesvirus 4	BFLF1	YP_401648.1	0.00E+00	31					
		Human herpesvirus 4	BFLF2	YP_401647.1	0.00E+00	18					
		Human herpesvirus 4	BFRF1A	YP_401728.1	2.20E-68	8					
		Human herpesvirus 4	BFRF1	YP_401649.1		12					
AJFE01025133.1	55674	Homo sapiens	uncharacterized protein	XP_002831604.2	6.00E-31	0.08	None				
		Papio anubis	Uronyl 2-sulfotransferase	XP_003919522.1	1.00E-30	8					
AJFE01035256.1	22558	Saimirine boliviensis	Uracil-DNA glycosylase	XP_003932325.1	7.38E-148	3	None				
		Pan paniscus	Uracil-DNA glycosylase	XP_003832558.1	2.00E-75	2					
AJFE01061695.1	31769	Gorilla gorilla	phoribosylformylglycinamidine synt	XP_004058598.1	8.00E-52	6	None				
		Homo sapiens	uncharacterized protein	XP_003846754.1	1.00E-39	5					
AJFE01068078.1	30507	Pan paniscus	uncharacterized protein	XP_003808117.1	2.00E-28	0.01	None				
		Otolemur garnettii	uracil-DNA glycosylase	XP_003789937.1	2.00E-62	0.02					
AJFE01002611.1	1196	Rhizobium sp	cyl-(acyl carrier protein) synthase II	WP_008535308.1	8.50E-01	0.2	None				
AJFE01002662.1	1003	Human herpesvirus 4	BWRF1	YP_401635.1	6.00E-11	0.2	None				
AJFE01080043.1	24258	Pan paniscus	uncharacterized protein	XP_003832743.1	2.00E-26	0.02	ORF virus	ORF127	NP_957904.1	1.00E-12	1
		cterized protein encoded by LINC0026	uncharacterized protein	XP_004092751.1	5.00E-33	0.03					
AJFE01100746.1	32067	Streptococcus agalactiae	hypothetical	WP_000321103.1	7.00E-115	0.06	[Lymphocystis disease virus	RNA-dependent DNA polymerase	YP_073558.1	8.00E-42	1
		Streptococcus agalactiae	Endonuclease	WP_000148030.1	0.00E+00	5					

Primary Hit							Alternative Hit				
Query ID	Length	Species	Description	Hit ID	E-value	coverage	Species	Description	Hit ID	E-value	Coverage
ABRT02259801.1	53666	Human herpesvirus 7	ssDNA-binding protein	YP_073781.1	3.00E-126	2%					
		Human herpesvirus 6B	transport/capsid assembly	NP_050221.1	0	4%					
		Human herpesvirus 6B	Glycoprotein B	NP_050220.1	0	4%					
		Human herpesvirus 6B	DNA polymerase	NP_050219.1	0	5%	Callithrix jacchus	DNA polymerase delta catalytic subunit	XP_002762441.1	6.00E-86	28%
		Human herpesvirus 7	DNA packaging protein UL32	YP_073776.1	3.00E-123	4%					
		Human herpesvirus 7	DNA packaging protein UL33	YP_073775.1	2.00E-26	4%	Sinorhizobium medicae	LPS:glycosyltransferase	WP_018209614.1	6.5	24%
		Human herpesvirus 6A	nuclear egress membrane protein	NP_042927.1	1.00E-59	1%					
		Human herpesvirus 7	protein UL49	YP_073773.1	2.00E-112	2%					
		Human herpesvirus 6B	large tegument protein	NP_050212.1	0	11%					
		Human herpesvirus 7	tegument protein UL37	YP_073770.1	3.00E-103	4%					
		Human herpesvirus 6A	capsid triplex subunit 1	NP_042922.1	1.00E-83	1%					
		Human herpesvirus 6B	large ribonuclease reductase	NP_050209.1	1.00E-151	1%					
		Human herpesvirus 7	DNA polymerase processivity subunit	YP_073767.1	2.00E-109	1%					
		Human herpesvirus 6B	putative membrane glycoprotein	NP_050201.1	5.00E-14	1%					
		Human herpesvirus 6B	immediate-early protein 4	NP_050199.1	2.00E-24	1%					
		Human herpesvirus 7	envelope glycoprotein UL37	YP_073758.1	1.00E-41	1%					
		Human herpesvirus 7	tegument protein vICA	YP_073757.1	6.00E-76	4%					
		Human herpesvirus 7	protein U15	YP_073756.1	6.00E-45	1%					
		Human herpesvirus 7	tegument protein UL35	YP_073755.1	3.00E-79	2%					
		Human herpesvirus 7	envelope glycoprotein UL33	YP_073753.1	3.00E-28	1%					
		Human herpesvirus 6B	antigenic virion protein	NP_050192.1	2.00E-47	1%					
		Human herpesvirus 6B	hypothetical protein HhV6Bgp016	NP_050191.1	2.00E-100	2%					
		Human herpesvirus 6A	protein UL28	NP_042898.2	0	7%					
		Human herpesvirus 6B	hypothetical protein HhV6Bgp009	NP_050186.1	2.00E-107	2%					
		Human herpesvirus 6A	tegument protein UL24	NP_042894.1	3.00E-47	2%					
		Human herpesvirus 6A	tegument protein UL23	NP_042893.1	6.00E-41	4%					
ABRT02337366.1	15583	Human herpesvirus 6B	IE-A transactivator	NP_050266.1	4.00E-11	9%	Clostridium sp. CAG:470	phosphoglycerate kinase	WP_022412440.1	2.1	2%
		Human herpesvirus 6B	Parvovirus rep homolog	NP_050269.1	2.00E-78	8%	Alistipes sp. CAG:53	uncharacterized protein	WP_022061947.1	2.2	3%
		Human herpesvirus 6A	protein U94/rep	NP_042987.1	6.00E-78	8%					
ABRT02391417.1	48621	Human herpesvirus 6A	protein DR6	NP_042888.2	1.00E-34	2%					
		Gibbon ape leukemia virus	Hypothetical mature RT protein	P_002000606	7.00E-25	2%	Chrysemys picta bellii	uncharacterized protein LOC101930828	XP_005294278.1	1.00E-25	2%
		Oreochromis niloticus	uncharacterized protein	P_005461302	2.00E-22	1%					
		Pongo abelii	UPF0764 protein C16orf89-like	P_003777030	4.00E-09	9%					
		Pongo abelii	histone demethylase UTY-like	P_003776766	6.00E-09	9%					
		Gorilla gorilla gorilla	histone demethylase UTY-like	P_004063026	1.00E-08	11%					
		Pongo abelii	UPF0764 protein C16orf89-like	P_003778043	3.00E-07	10%					
ABRT02275240.1	34290	Human herpesvirus 6B	Helicase/primase complex	NP_050256.1	9.00E-164	7%	Pseudoalteromonas agarivorans	hypothetical protein	WP_004589690.1	1.00E-41	2%
		Human herpesvirus 6B	major immediate-early protein	NP_050265.1	6.00E-77	6%					
		Human herpesvirus 6B	Glycoprotein L	NP_050261.1	2.00E-27	2%					
		Human herpesvirus 7	protein UL117	YP_073821.1	2.00E-38	1%					
		Human herpesvirus 7	uracil-DNA glycosylase	YP_073819.1	9.00E-61	2%	Trichomonas vaginalis G3	uracil-DNA glycosylase family protein	XP_001296939.1	8.00E-27	2%
		Cyanothece sp. CCY0110	hypothetical protein, partial	P_008278991	6.00E-94						
		Human herpesvirus 6B	putative viron protein	NP_050255.1	3.00E-152	5%					
		Human herpesvirus 6A	helicase-primase helicase subunit	NP_042970.1	3.00E-137	7%					
		Human herpesvirus 7	DNA replication origin-binding helicase	YP_073813.1	2.00E-111	6%					
		Human herpesvirus 7	envelope glycoprotein M	YP_073812.1	3.00E-119	2%					
		Human herpesvirus 6A	deoxyribonuclease	NP_042963.1	2.00E-109	4%					
		Human herpesvirus 6B	tegument protein	NP_050245.1	1.00E-99	2%					
		Human herpesvirus 6A	protein UL95	NP_042960.1	1.00E-57	2%					
		Human herpesvirus 6B	Putative terminase	NP_050241.1	6.00E-79	2%					
		Human herpesvirus 6B	tegument protein	NP_050245.1	1.00E-99	2%	Glycine max	lectin S-receptor-like serine/threonine-protein kinase At2g191	XP_003547437.1	0.1	1%
ABRT02394679.1	5129	Ceratotherium simum simum	uracil-DNA glycosylase	P_004429930	1.00E-134	16%					
ABRT02359611.1	17917	Pongo abelii	UPF0764 protein C16orf89-like	P_003777030	6.00E-14	30%					
		Homo sapiens	histone demethylase UTY isoform X3	P_005262577	9.00E-06	26%					
		Pan paniscus	lecule in BECN1-regulated autophagy protei	P_003815253	0.13	27%					
		Macaca fascicularis	chemokine (C-C motif) receptor 7 precursor	P_001272026	0	6%					
ABRT02400840.1	8590	Pan troglodytes	NA polymerase delta catalytic subunit, partia	P_003316601	8.00E-35	17%					
ABRT02455554.1	25188	Nomascus leucogenys	uncharacterized protein encoded by LINC00	P_004092751	2.00E-12	24%					
		Nomascus leucogenys	uracil-DNA glycosylase-like	P_003278500	3.00E-27	6%					
